# Spatially localized fMRI metrics as predictive and highly distinct state-independent fingerprints

**DOI:** 10.1162/IMAG.a.1444

**Published:** 2026-11-06

**Authors:** Daouia I. Larabi, Martin Gell, Enrico Amico, Simon B. Eickhoff, Kaustubh R. Patil

**Affiliations:** Institute of Neuroscience and Medicine, Brain & Behaviour (INM-7), Research Centre Jülich, Jülich, Germany; Institute of Systems Neuroscience, Medical Faculty, Heinrich Heine University Düsseldorf, Düsseldorf, Germany; Department of Clinical and Developmental Neuropsychology, University of Groningen, Groningen, The Netherlands; Department of Psychiatry, Psychotherapy and Psychosomatics, Medical Faculty, RWTH Aachen University, Aachen, Germany; School of Mathematics, University of Birmingham, Birmingham, UK; Centre for Human Brain Health, University of Birmingham, Birmingham, UK

**Keywords:** fMRI, functional connectivity, regional homogeneity, cognition, Human Connectome Project, machine learning

## Abstract

Precision medicine and the investigation of brain–behavior associations require biomarkers that are stable (low intraindividual variability) and unique (high-interindividual variability) at the same time, hence calling them “fingerprints”. The functional connectome (FC) has good “fingerprint properties”, as individuals can be accurately identified in a database based on their FC. Importantly, research has shown lower intraindividual variability of more localized measures of brain function, such as regional homogeneity (ReHo) and (fractional) amplitude of low-frequency fluctuations ((f)ALFF), compared with the FC. Additionally, the predictiveness of behavior using the FC is modest at best. Here, with fMRI data from two publicly available datasets, we demonstrate that individuals can be identified with near-perfect accuracies using local functional fingerprints, especially the regional homogeneity (ReHo) fingerprint. Further analyses reveal that the dorsal attention network contributes most to the individual “uniqueness” of the ReHo fingerprint. Moreover, using a machine learning setup, we find that intraindividual variability of the ReHo fingerprint across sessions shows predictive signal for individual-level intelligence, albeit with model performance dependent on the condition. Last, using two other publicly available datasets, clinical applicability is demonstrated with high fingerprint accuracies and a significant correlation between fingerprint stability and intelligence in individuals with schizophrenia. Altogether, our findings suggest that the ReHo fingerprint is a good candidate for further exploration of applicability in precision medicine.

## 1 Introduction

Large variability across individuals (i.e., interindividual variability) is seen in phenotypic traits, including physical characteristics, cognitive abilities, and brain organization. Identifying the interindividual variability in functional brain organization that is predictive of cognitive and clinical properties at the individual level is essential for developing viable biomarkers for precision medicine and *precision psychiatry* specifically (Fernandes et al., 2017). Precision psychiatry is the tailoring of treatment to the individual. It requires trait-like representations of brain function that are simultaneously reliable (i.e., show low *intraindividual* variability) and unique (i.e., show high *interindividual* variability), that is, stable representations that vary across individuals. Intraindividual stability independent of an individual’s current state is a prerequisite for clinical application, as biomarkers should reflect underlying traits or disease and not the current state. More specifically, they should be stable from day to day or for a few months at maximum, as they should be resilient to non-meaningful fluctuations (e.g., having a bad day) but sensitive to clinical fluctuations (e.g., severe low mood in depression). Representations should also be unique for an individual, as the interindividual variability in these representations can then be utilized to predict individual-level behavior with machine learning algorithms (J. Chen et al., 2020). Thus, a trait-like state-independent neural fingerprint that captures interindividual differences can illuminate fundamental brain organization and facilitate translational applications.

Until now, the functional connectome (FC) has been *the* choice to predict cognition and behavior. It reflects whole-brain patterns of synchrony in fluctuations of the blood-oxygen-level-dependent (BOLD) signal, as measured with functional magnetic resonance imaging (fMRI). The FC is usually calculated as Pearson’s correlations between the time courses of all possible pairs of regions. It has been termed a functional connectome “fingerprint” as it is highly stable and sufficiently unique to identify an individual across sessions (Finn et al., 2015; Seitzman et al., 2019). The FC has also been shown to capture interindividual differences (Finn & Rosenberg, 2021; Nostro et al., 2018): the variability in the FC fingerprint has been associated with development, aging, intelligence, and psychiatric disorders (Amico & Goñi, 2018; Kaufmann et al., 2017, 2018; Miranda-Dominguez et al., 2014; Noble et al., 2019). It can specifically indicate disorders such as schizophrenia, depression, or attention deficit disorder (Kaufmann et al., 2018). Hence, it fulfills the previously mentioned properties of low intraindividual variability, and high interindividual variability subsequently called “fingerprint properties” in this manuscript.

However, even though the FC is most commonly used for the prediction of cognitive and clinical properties, several studies have shown lower test–retest reliability of global measures of brain function as opposed to local measures such as regional homogeneity (ReHo), amplitude of low-frequency fluctuations (ALFF), and fractional ALFF (fALFF) (B. Chen et al., 2015; Holiga et al., 2018; Jiang & Zuo, 2016). Specifically, local metrics show lower intraindividual variability, suggesting that these metrics are less dynamic than global metrics (B. Chen et al., 2015). Therefore, here, we empirically compared the FC baseline “fingerprint” with local functional “fingerprints” within predefined brain regions based on the 264 Power coordinates (Power et al., 2011). We quantified fingerprint properties within the identification framework, in which identification is the ability to identify an individual in a new database. We used the resting-state and task fMRI data of the Human Connectome Project (HCP) and four different ways to quantify identification, including the proposed novel scores “ranking accuracy” and “forensic identification accuracy”. We found near-perfect identification accuracies when using ReHo, followed by ALFF, fALFF, and the FC. We extensively checked for replicability of these findings across various parcellations and resilience against confounding effects. Next, we conducted four follow-up analyses. First, using a novel optimization formulation, we investigated which regions contributed most to making the ReHo fingerprint “unique” at the individual level. Second, the previously mentioned fingerprint properties of reliability and uniqueness are necessary but insufficient for clinical applicability, given that fingerprints should also capture interindividual variance related to actual behavior (Finn & Rosenberg, 2021; Mantwill et al., 2021). As previous studies have shown more stable FC patterns across resting-state and task sessions in individuals with higher intelligence (Schultz & Cole, 2016), we tested this by using both the fingerprints themselves and the *fingerprint stability*—that is, their invariance across sessions—to predict crystallized and fluid intelligence in a machine learning setup. This allowed us to test whether the intraindividual fingerprint stability between sessions captures individual-level variance in cognitive properties. Note that “sessions” refers to the different task-based and resting-state fMRI data sessions, which are acquired within a single or in multiple visits. Third, to demonstrate further clinical applicability in a clinical sample, we investigated fingerprints and the correlation between fingerprint stability and intelligence in two samples with individuals with a psychotic disorder and healthy controls, namely the HCP Early Psychosis (HCP EP) sample and OpenNeuro.ds000115 sample (Repovš & Barch, 2012). Finally, to address the question of neural origins of the BOLD signal (Arthurs & Boniface, 2002), using data from the enhanced Nathan Kline Institute (eNKI) sample, we compared fingerprints between rs-fMRI and breath-holding scans that are known to capture cerebrovascular signals (Pinto et al., 2020). Our findings suggest that local neural activity measures, and especially ReHo, show high specificity, sensitivity, and robustness as a neural fingerprint.

## 2 Methods

### 2.1 Datasets and preprocessing

The following datasets were used: Human Connectome Project Young Adult (HCP YA) S1200 release (https://www.humanconnectome.org/study/hcp-young-adult), OpenNeuro.ds000115 (Repovš & Barch, 2012) (https://openneuro.org/datasets/ds000115/versions/00001), HCP Early Psychosis (HCP EP) Release 1.1 (https://www.humanconnectome.org/study/human-connectome-project-for-early-psychosis), and enhanced Nathan Kline Institute (eNKI) dataset (Nooner et al., 2012) (http://fcon_1000.projects.nitrc.org/indi/enhanced/). Details of these datasets including additional preprocessing steps are described in the Supplementary Materials. Preprocessing included regressing out the 12 detrended motion parameters provided by the HCP (Movement_Regressors_dt.txt) to correct for head motion. Written informed consent was obtained from all participants for being included in the study.

### 2.2 Analyses

Code for the main analyses is available on GitHub (https://github.com/juaml/local_fc_fingerprint).

#### 2.2.1 Local fingerprints

A local fingerprint for a subject consists of a vector of length N, where N is the number of ROIs. ReHo is calculated with Kendall’s coefficient of concordance considering the time series of all the voxels within an ROI (Y. Zang et al., 2004). For the main analyses, ReHo was calculated using the voxels within a 3-mm sphere around 264 coordinates. For ALFF and (f)ALFF, voxel-wise time series were transformed to the frequency domain to obtain the power spectrum (Zou et al., 2008). ALFF is the average square root of the power in the 0.01–0.1 Hz frequency range averaged across voxels within an ROI (Y.-F. Zang et al., 2007). fALFF is ALFF scaled by the total power in the full frequency range averaged across voxels within an ROI (Zou et al., 2008). ALFF and fALFF were calculated per voxel and consequently averaged across voxels within an ROI.

#### 2.2.2 Global fingerprint (whole-brain functional connectome)

An FC for a subject consists of a matrix of size N x N, where N is the number of ROIs. We extracted the lower triangle of this matrix and vectorized it to obtain the FC fingerprint as a vector of length N x (N−1)/2. The FC fingerprint is larger in size than local fingerprints, which might be a disadvantage for further analyses. Therefore, we additionally performed PCA on the FC fingerprint to obtain a “PCA FC fingerprint” consisting of a vector of length N. This fingerprint was obtained by applying PCA to the resting-state 1 session data after averaging the LR and RL phase-encoding directions. Subsequently, components were estimated in the same space for resting-state 2 and task sessions.

#### 2.2.3 Identification accuracy

Iteratively, all subjects’ data from one session were used as the “base session” while data from another session were used as the “target session”. Spearman correlations were calculated between the fingerprints of the two sessions. Using a 1-nearest neighbor approach (Finn et al., 2015), the “base” fingerprint was labeled with the subject identity of the most similar fingerprint in the “target session”. Identification accuracy was calculated as the fraction of correctly labeled participants. Identification accuracy was calculated for all fingerprints, including the additional PCA FC fingerprint.

#### 2.2.4 Differential identifiability (Idiff)

An identification matrix of size NxN (N = number of subjects) was created reflecting the correlations between the fingerprints of a subject in the base and all fingerprints in the target database. The lower triangle elements were extracted and represented as an N x (N-1)/2-dimensional vector. “Iself” is the average within-subject similarity, while “Iothers” is the average between-subjects similarity. Idiff is then calculated by taking the difference between Iself and Iothers (Amico & Goñi, 2018). Idiff was calculated for all fingerprints, including the additional PCA FC fingerprint.

#### 2.2.5 Ranking accuracy

Identification accuracies were calculated (see paragraph “Identification accuracy” above) with one difference. Namely, iteratively, each session was used as the base session, while all other eight sessions (instead of only one other session) were included in the target session. Then, instead of focusing only on the fingerprint in the target session with the highest correlation, all fingerprints in the target session were ranked based on their similarity with a subject’s fingerprint in the base session. At each rank (2–9, as each subject has data from nine sessions), the proportion of correctly identified subjects was calculated (see Fig. 1 for a visualization). Ranking accuracy was calculated for all fingerprints, including the additional PCA FC fingerprint.

**Fig. 1.**
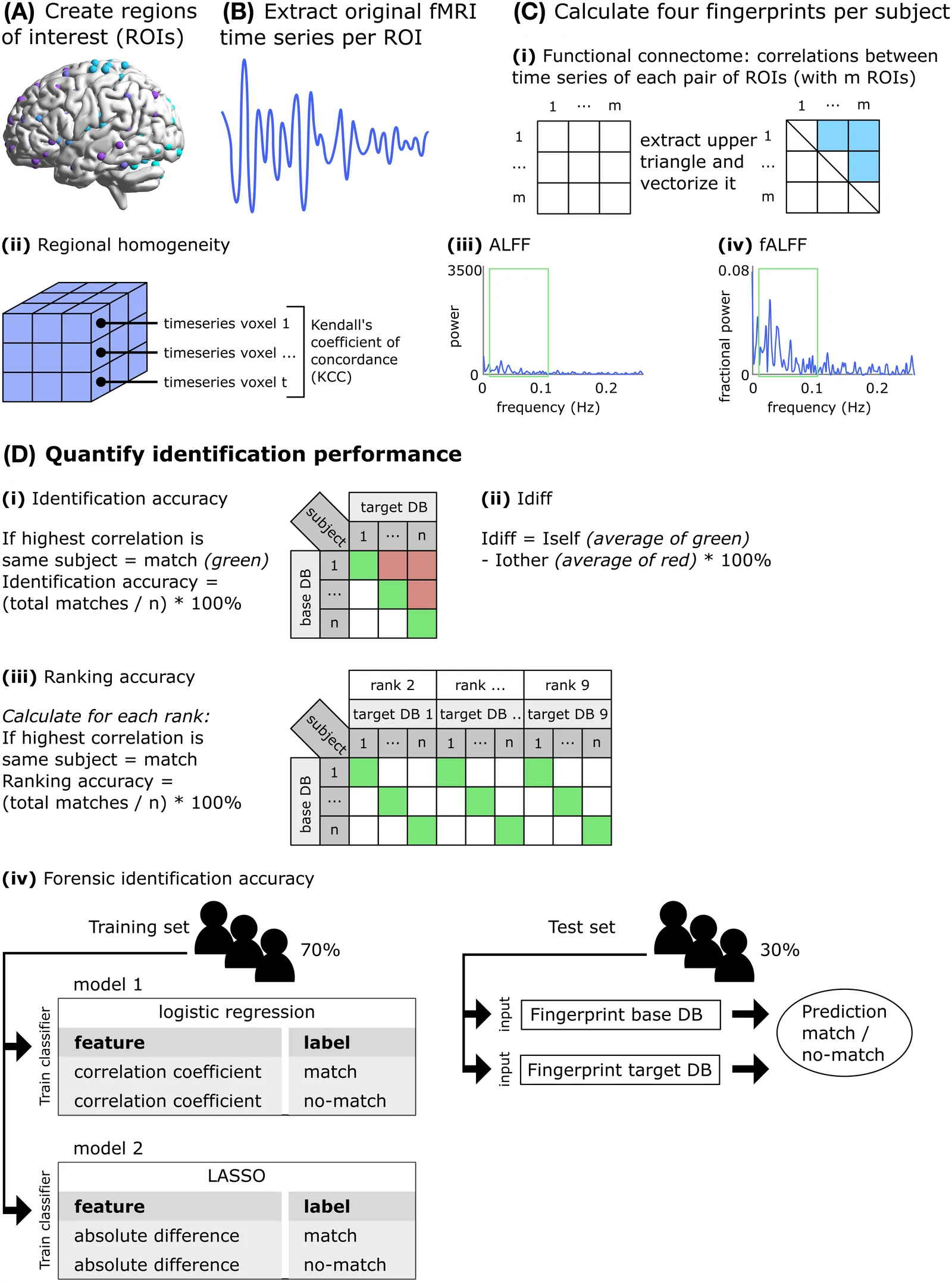
Schematic representation of identification methods. (A) Creation of regions of interest (ROIs). (B) Extraction of original fMRI time series per voxel or ROI. ReHo was calculated using Kendall’s coefficient of concordance (KCC) across the time series of all voxels within each ROI. ALFF and fALFF were calculated per voxel and then averaged across voxels within each ROI. For the functional connectome, time series were extracted directly per ROI. (C) Calculation of four fingerprints per subject: functional connectome, regional homogeneity (ReHo), amplitude of low-frequency fluctuations (ALFF), and fractional ALFF (fALFF). (D) Quantification of identification performance using identification accuracy, differential identifiability (Idiff), ranking accuracy, and forensic identification accuracy.

#### 2.2.6 Forensic identification

We used a machine learning framework to predict whether two fingerprints matched (see Fig. 1). For this, we used the two resting-state sessions as training data and trained a classification model using a match-no-match dataset derived from 70% of the subjects (randomly chosen). Forensic identification accuracies were calculated across all pairs of sessions. We tested two different feature spaces that were constructed containing a match and a no-match part. The match part contained features of fingerprints from two sessions of the same subject. The no-match part contained features of a subject from session 1 and their second-closest match from session 2. For the first model, the feature space included (for each subject) the correlation coefficient between two resting-state fingerprints. For the second model, the features for the match part included (for each subject) the absolute difference between the elements of two resting-state fingerprints. Similarly, the features for the no-match part included the absolute difference between the resting-state 1 fingerprint of a subject in the base session and the resting-state 2 fingerprint of the closest other subject in the target session. As the feature space is either the correlation between fingerprints (feature space of 1) or the absolute difference between elements (feature space of #regions), the resting-state sessions were chosen for training as both are smallest with the most similar fingerprints, that is, with the RS fingerprints. This provided a dataset with 2xN rows (N = number of subjects), which, together with class labels indicating match or no-match, was used to train a classifier. More specifically, we employed logistic regression with the feature space of the first model and LASSO with the feature space of the second model. The 30% hold-out subjects were used for testing: each of the 30% held-out subjects was probed against all the subjects one by one as a reference to obtain matches. Given a reference fingerprint (with known identity) and a test fingerprint (unknown identity), a trained model was used to predict whether they matched. A prediction was then obtained using each fingerprint in a reference dataset. The reference dataset was either the average of the two resting-state sessions or one of the task sessions (emulating the privacy-preserving scenario where the training data are unavailable). As the number of predictions for each test fingerprint equals the number of fingerprints in the reference dataset, predictions for a test fingerprint can contain one, multiple, or no matches. If the correct subject was predicted as a match, it was counted as a true positive (TP), and if any other subject was predicted as a match (irrespective of a TP), then this was counted as a false positive (FP). A good classifier should provide a high TP and a low FP rate. The split between training and test subjects was repeated 10 times. Ten times repeated 10-fold cross-validation was performed on the training data to estimate the accuracy on a hold-out test set, used as a proxy for measuring generalization ability (Scheinost et al., 2019).

#### 2.2.7 Confound removal

Confounding signals due to age, sex, and total intracranial volume (ICV) were regressed out from each column of each database (subjects x fingerprint), and the residuals were used for identification. The ICV was estimated using the CAT12.5 VBM processing pipeline.

#### 2.2.8 Contribution of regions to individual uniqueness via margin optimization

To identify important ROIs whose ReHo is individual-specific, we posed an optimization problem to derive weights for the weighted Pearson correlation coefficient that maximizes the margin, that is, the correlation difference between the correct fingerprint and the next best. As the ReHo identification accuracy was very high (close to 100% in most cases), using the accuracy as an optimization objective would not be meaningful. We sought to identify a weight w_i_ for each ROI by solving the following optimization problem:

$$argmax_{w}\sum_{sub}\;\varrho_{w}\left(self\right)-\;\varrho_{w}\left(best_{\neq self}\right)$$

$$s.t.\;w_{i}\in \left[0,1\right].$$

Here we are looking for the value of w that maximizes (argmax) the following expression: summed over subjects (∑sub), the difference between the correlation between the two fingerprints of the same subject ($\varrho_{w}$ (self) and the correlation between a fingerprint and the next best fingerprint $\varrho_{w}$(best≠_self_) such that w_i_ falls between 0 and 1$\;\left(s.t.\;w_{i}\in \left[0,1\right]\right)$. This optimization problem was solved to maximize the average margin for the HCP sample (n = 399) using the CMA-ES algorithm (Hansen & Ostermeier, 2001). Importantly, the data were divided into a 50% training set used to learn the weights and a 50% test set to validate whether the optimized weights indeed improve the margin on out-of-sample data. This procedure was repeated 20 times to assess stability. We used two optimization problems: RS1-RS2 and RS2-Motor. This resulted in an importance weight for each ROI, reflecting its contribution to uniqueness (Supplementary Figs. S1 and S2). Next, each of the Power 3-mm ROIs was assigned to one of the seven Yeo networks or “None” based on the mode of the network membership of the voxels within each ROI (Supplementary Table S1). Then, we varied a threshold over the optimized weights (averaged over RS1-RS2 and RS2-Motor) to assess the retained proportion of the ROIs from each of the seven networks.

#### 2.2.9 Fingerprints and fingerprint stability predict intelligence

The fingerprints were used to predict individual-level crystallized (“CogCrystalComp_AgeAdj”) and fluid intelligence (“CogFluidComp_AgeAdj”) separately in a five times repeated fivefold cross-validation setup using least absolute shrinkage and selection operator (LASSO) regression (Hastie et al., 2009). The variance due to age and total intracranial volume was removed from the features in a cross-validation consistent manner (More et al., 2021). Additional analyses were conducted in which variance attributed to head motion was further removed by including mean frame-wise displacement (averaged within each session) to control for head motion-related effects.

Next, to investigate the predictiveness of fingerprint *stability*, we first calculated—for each individual—the stability of the fingerprints as the average Pearson correlation of each session’s fingerprint with the fingerprints of all other sessions resulting in a feature vector with nine values (i.e., two resting-state and seven task fMRI sessions). These features were then employed for prediction analysis as described above.

To ensure stability of the results across machine learning algorithms, we additionally used the fingerprints and fingerprint stability to predict intelligence in a five times repeated fivefold cross-validation setup using Elastic Net.

Note that the PCA FC fingerprint nor its stability was used for predicting intelligence. As this fingerprint needs a reference, it needs a reference dataset as well as a scheme to use it in a machine learning setup without causing data leakage. Further work is needed for properly designing such analyses that are out of the scope of the current study.

## 3 Results

Analyses were performed using data from 399 unrelated subjects with 2 resting-state and 7 task-based scans (acquired within 2 days) from the S1200 release of the HCP YA sample (Feinberg et al., 2010; Moeller et al., 2010; Setsompop et al., 2012; Van Essen et al., 2013; J. Xu et al., 2021). The brain was parcellated into 264 spherical regions with a 3-mm radius around the Power coordinates (Power et al., 2011) (as analyses showed that smaller regions of interest (ROIs) yielded better identification accuracies—Supplementary Fig. S3). The global FC fingerprint was calculated as the Pearson correlations between the mean time series of each pair of ROIs (Finn et al., 2015) (Fig. 1). Furthermore, three local fingerprints were calculated: ReHo (Y. Zang et al., 2004), ALFF (Y.-F. Zang et al., 2007), and fALFF (Zou et al., 2008). Each of these measures captures distinct aspects of intrinsic brain function: the often-reported FC fingerprint reflects the similarity between the mean time series of each pair of spatially distant ROIs (i.e., interregional similarity), while ReHo reflects the synchronization of the voxels’ time series within an ROI (i.e., intraregional similarity), and (f)ALFF reflects BOLD fluctuations within an ROI (i.e., amplitude of intraregional activity). Next, identification accuracies, that is, the fraction of correctly identified subjects, were calculated across all pairs of sessions. We observed perfect identification accuracies of 100% between the two resting-state sessions for all fingerprints. Moreover, identification accuracies were perfect across task–task and resting-state–task combinations when using ReHo (mean: 100%), followed by ALFF (96%), fALFF (92%), and FC (84%) (Fig. 2A), suggesting low intraindividual variability and high individual specificity. However, differences between the FC fingerprint and local fingerprints may be influenced by differences in dimensionality as the FC fingerprint is of larger size (see section Similarly sized FC fingerprint). Note that we did not perform statistical comparisons of identification accuracies; rather, we present numerical differences between fingerprints, consistent with previous studies (Amico & Goñi, 2018; Finn et al., 2015). Next, we investigated whether our findings hold for different identification methods.

**Fig. 2.**
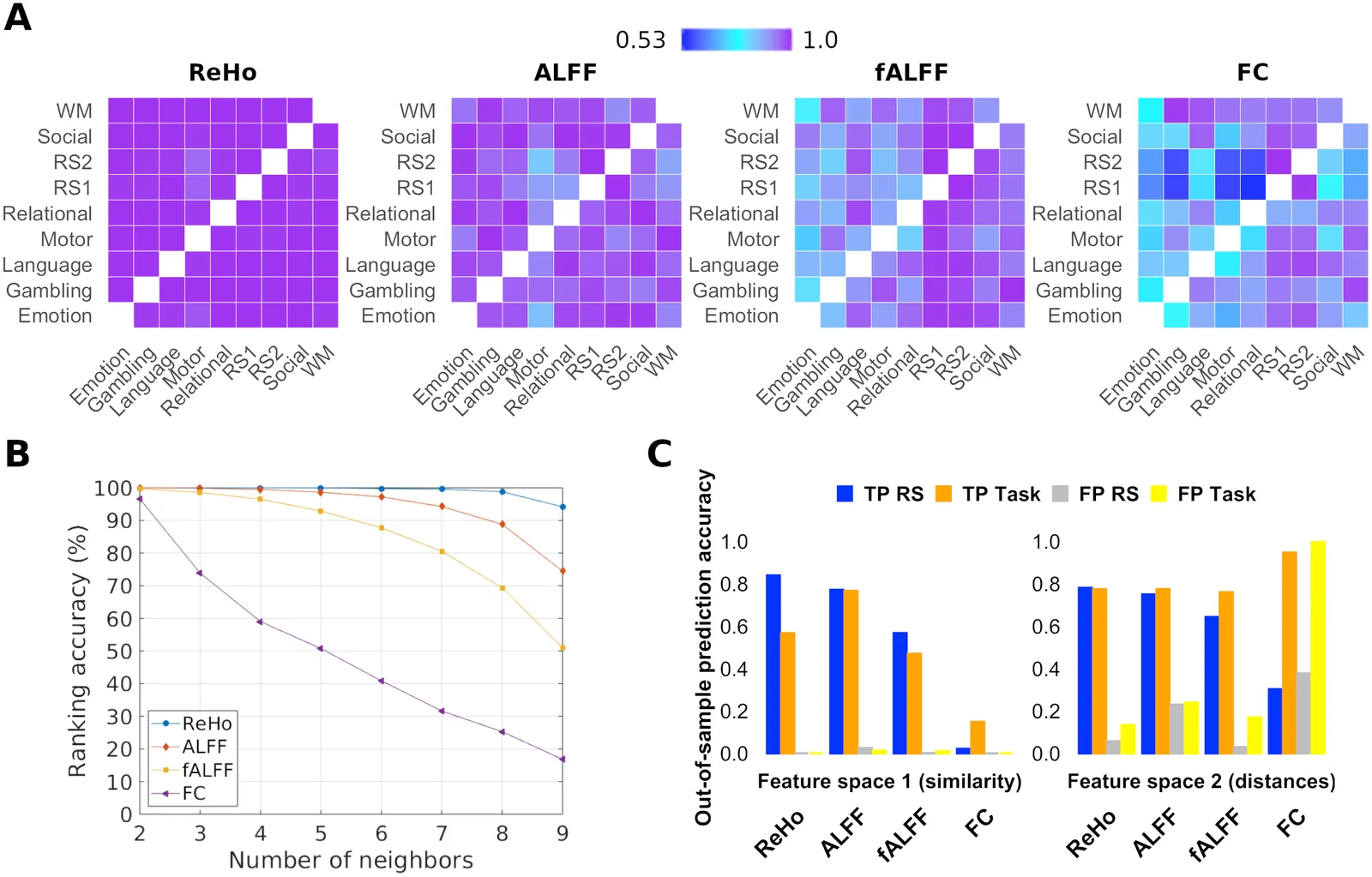
(A) Comparison of identification accuracies of fingerprints in the HCP Young Adult sample. (B) Comparison of identification accuracies based on the ranking of fingerprints in the HCP sample. Note that at rank 2, the ranking accuracy is similar to the conventional identification accuracy, where the FC fingerprint performs relatively similar to the local fingerprints. Still, the difference between them becomes evident at higher ranks. (C) Forensic identification performance using fingerprint similarity as a feature (feature space 1; using logistic regression) or the pattern of distances between the fingerprint elements as features (feature space 2; using LASSO). For the FC, the nearly zero true-positive and false-positive rates with feature space 1 (fingerprint similarity; left of Fig. 2C) indicate that it failed to match any of the test subjects. The high true-positive and false-positive rates with feature space 2 (pattern of distances between fingerprint elements; right of Fig. 2C) indicate that it matched many fingerprints correctly (true positives) but also incorrectly (false positives). ReHo = regional homogeneity; ALFF = amplitude of low-frequency fluctuations; fALFF = fractional ALFF; FC = functional connectome; RS = resting state; TP = true-positive rate; FP = false-positive rate; HCP = Human Connectome Project.

### 3.1 Highly accurate individual identification based on local functional fingerprints irrespective of identification performance quantification

First, we quantified the identification performance using the often-reported differential identifiability (“Idiff”) score. This score is the difference between a fingerprint’s average within-subject (“Iself) and average between-subject similarity (“Iothers”); a higher Idiff indicates a better fingerprint (Amico & Goñi, 2018). Idiff scores were consistent with the accuracy results. Idiff between the two resting-state sessions is highest for ReHo (69), followed by ALFF and fALFF (both 56), and the FC (29). The highest Idiff for resting-state–task and task–task combinations was found for ReHo (mean: 52), followed by ALFF (47), fALFF (38), and FC (9) (Supplementary Fig. S4; for general interest, additional results using a second parcellation (Dosenbach et al., 2010) are also reported). To provide further insight, we also show boxplots of Iself and Iothers separately in Supplementary Fig. S5 and performed statistical comparisons between them, confirming that within-subject similarity is significantly greater than between-subject similarity.

For those two conventional quantifications of identification performance, it is assumed that there is one fingerprint of a subject in the base database and one in the target database, and only the most similar fingerprint is considered a match. Here, we explored two alternative scenarios that relax these assumptions. Importantly, the first scenario still operates under a controlled setting in which multiple fingerprints per subject are present in the target database, with the number of samples per subject fixed in advance. We refer to this evaluation as “*ranking accuracy*”, which quantifies the degree to which fingerprints of *all subject sessions* are consistently ranked higher (i.e., show higher identification accuracy) than fingerprints of other subjects. This stricter measure yielded the same pattern of results (Fig. 2B). In contrast, real-world forensic applications typically involve uncertainty in whether and how many fingerprints from the same subject are present in the database. To address this more realistic setting, we additionally evaluated a “forensic identification” scenario that allows for the possibility of a “no-match”. The results clearly demonstrate the superiority of the local fingerprints (Fig. 2C).

### 3.2 Similarly sized FC fingerprint

The FC fingerprint is of larger size (i.e., N x N regions) than the local fingerprints (i.e., N regions). To check the effect of the size of the FC fingerprint on quantification of identification, we created an FC fingerprint of similar size as the local fingerprints. Therefore, we performed PCA on the FC fingerprint to obtain a “PCA FC fingerprint” consisting of a vector of length N. To obtain this fingerprint, PCA was done on data of resting-state 1 session (averaged for LR- and RL-phase encodings). Subsequently, fingerprints were estimated for resting-state 2 and task sessions by projecting the corresponding FC in the PCA space of RS1. Similar to the other fingerprints, identification accuracies between the resting-state sessions were 100% (see Supplementary Fig. S6 for a comparison with the other fingerprints). For the resting-state–task and task–task combinations, the mean identification accuracy of 99% was lower than for the ReHo fingerprint, but higher than for the other fingerprints. Idiff between the two resting-state sessions was highest for the PCA FC fingerprint (81; second-best fingerprint ReHo: 69; see Supplementary Fig. S7). This dropped to 43 for resting-state–task and task–task combinations, making it perform poorer than the ReHo and ALFF fingerprint. The ReHo and ALFF fingerprint also outperform the PCA FC fingerprint on ranking accuracy (Supplementary Fig. S6B). Note that forensic identification accuracy was not calculated for the PCA FC fingerprint. This would require the definition of a reference PCA space for which there are no clear guidelines available; therefore, defining one would be out of the scope of this study.

Importantly, the PCA-based FC fingerprint, which reduces the original N x N connectivity matrix to an N-dimensional representation, achieved higher identification performance than the full FC representation. This suggests that differences in fingerprint performance are partly related to differences in representational dimensionality. Consequently, comparisons between FC-based and local fingerprints should be interpreted in light of these differences, rather than as purely reflecting differences in underlying signal specificity.

### 3.3 Better identification performance of local functional fingerprints is replicable across parcellations and samples and resilient to confounding effects

Additionally, we performed extensive evaluations to establish replicability and specificity of our results obtained with the Power 3-mm ROIs. First, we tested three other parcellations, namely, Dosenbach (Dosenbach et al., 2010), Shen (Shen et al., 2013), and Schaefer with 300 ROIs (Schaefer et al., 2018), which also showed higher identification accuracies for the local fingerprints (Supplementary Fig. S8). Similar to our results with the Power spheres, the Dosenbach parcellation showed the highest identification accuracies for task–task and resting-state–task combinations for ReHo (94%), followed by ALFF (92%), fALFF (79%), and FC (77%). Identification accuracies between the resting-state sessions were 100% for ReHo and fALFF, and 99% for ALFF and FC. With the Shen and Schaefer parcellations, the results followed a similar pattern with a slight difference in the order: the highest identification accuracies were found for ALFF (77% and 79%), followed by ReHo (67% and 62%), FC (51% and 51%), and fALFF (35% and 46%). Using the Shen parcellation, identification accuracies between the resting-state sessions were highest for ReHo (98%), followed by FC (96%), ALFF (94%), and fALFF (91%). For the Schaefer parcellation, identification accuracies between resting-state sessions were highest for FC (98%), followed by ReHo (95%), fALFF (94%), and ALFF (93%). Second, we investigated the effect of removing confounds, that is, sex, age, and total intracranial volume, from the fingerprints. Removing these confounds increased overall accuracy while retaining the result pattern—the highest accuracy with ReHo (Supplementary Fig. S9). Last, replication on the smaller Finn et al. (2015) HCP sample (n = 125) showed similar results with some minor differences (see Supplementary Materials for details; Figs. S10 and S11).

### 3.4 The dorsal attention and frontoparietal networks contribute most to individual uniqueness

Some brain networks contribute more to the “uniqueness” of the FC fingerprint than others (Finn et al., 2015; Jo et al., 2020). Encouraged by the high accuracy of the ReHo fingerprint, we investigated the contribution of each ROI in making the ReHo fingerprint unique. Each region was assigned to one of the seven Yeo networks based on the mode of the network membership of the voxels within that ROI (Yeo et al., 2011). Since identification accuracies were highest between the resting-state sessions but lowest with the motor task (Finn et al., 2015), data from these three sessions were used. We found that the ROIs contributing most to uniqueness were located in the dorsal attention network, while the limbic network contained the fewest individual-specific ROIs (Fig. 3).

**Fig. 3.**
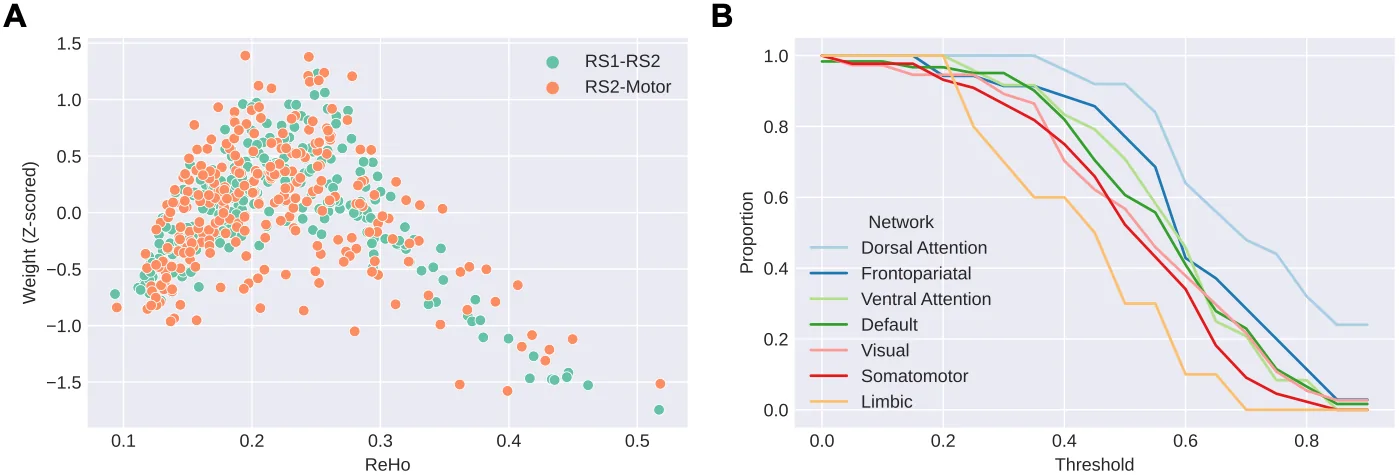
Contribution of regions in making the ReHo fingerprint individual specific. Weights for a weighted Pearson correlation were optimized to maximize the margin between the correct fingerprint and the next best, yielding an importance weight for each ROI. This reflected its contribution to uniqueness. ROIs were assigned to one of Yeo’s seven networks based on the mode of voxel membership. Thresholding the optimized weights (averaged over RS1–RS2 and RS2–Motor) revealed the proportion of retained ROIs per network. (A) Relationship between ReHo and Z-scored importance weights of regions averaged across all optimization runs. The average ReHo across subjects shows an inverse U-shaped relationship with the weights, with higher weights indicating higher individual uniqueness. (B) The proportion of regions in Yeo’s seven networks. ReHo = regional homogeneity; RS1 = resting-state fMRI session day 1; RS2 = resting-state fMRI session day 2; Motor = fMRI motor task.

### 3.5 Fingerprints and fingerprint stability capture interindividual differences

Next, for real-world and especially clinical applications, representations of brain function should not only have good fingerprint properties but should also capture interindividual differences in behavior or cognition. Interestingly, earlier studies have shown that the variation in the FC fingerprint measured across fMRI sessions, thus reflective of (task-related) reconfiguration, is lower in individuals with higher intelligence (Schultz & Cole, 2016; Thiele et al., 2021). Therefore, we chose to predict intelligence.

The prediction analyses were designed as a structured benchmarking framework rather than a set of independent hypothesis tests. Specifically, we evaluated a predefined set of regression models (i.e., LASSO and elastic net), imaging conditions (i.e., task and resting state), feature types (i.e., FC, ReHo, fALFF, and ALFF), and prediction targets (i.e., fluid and crystallized intelligence) to assess the relative performance and robustness of fingerprint-based representations. Accordingly, inference in this context is based on consistency of effects across models, datasets, and feature representations rather than on significance tests. We, therefore, interpret results in terms of stability of patterns across the analyses. A prediction was considered successful if (i) the median prediction accuracy, quantified as the Pearson correlation between predicted and observed values, exceeded a threshold of 0.15 and (ii) consistent performance was observed across both LASSO and Elastic Net models. This dual criterion was used to ensure robustness across models.

First, we investigated the use of fingerprints to predict intelligence in the HCP dataset using two different ML algorithms: LASSO and Elastic Net. The variance due to age and total intracranial volume was removed from the features in a cross-validation consistent manner (More et al., 2021). We found a pattern with the local fingerprints being more predictive than the FC fingerprint: ReHo of fluid intelligence using the Language fMRI task (r = 0.16–0.18); ALFF of fluid intelligence using the Motor fMRI task (r = 0.16–0.2), and fALFF of crystallized (r = 0.17) and fluid intelligence (r = 0.18–0.19) using the Motor fMRI task (Supplementary Fig. S12). These effects were modest and varied across tasks and feature types. As prior work has demonstrated that head motion is associated with intelligence (Siegel et al., 2017) and can influence fMRI-derived measures, we additionally report results in which variance related to average frame-wise displacement (averaged across timeframes of one session) was regressed out from the features in a cross-validation–consistent manner. After removing head-motion–related variance, the prediction of fluid intelligence from ReHo using the Language task remained (r = 0.14), but no longer met the predefined criterion for successful prediction (median r > 0.15). In contrast, the previously observed predictions based on ALFF and fALFF during the Motor task were no longer present (Supplementary Fig. S13).

Next, in addition to previous studies using only the fingerprint itself to predict cognition, we also examined the use of *the stability of the fingerprints* for the prediction of intelligence. Earlier studies have shown smaller changes in FC patterns between resting state and tasks in individuals with higher intelligence (Schultz & Cole, 2016), as well as more efficient (i.e., smaller amplitude) task-evoked brain activations (Neubauer & Fink, 2009). This begs the question whether the intraindividual fingerprint stability across sessions is still meaningful for explaining cognitive properties at the individual level. This was tested by using the stability of the fingerprints to predict intelligence. We observed that the stability of ReHo was predictive of crystallized intelligence (r = 0.2), and ALFF stability predicted both crystallized (r = 0.18) and fluid intelligence (r = 0.18). In contrast, the stability of fALFF and FC was generally not predictive (Fig. 4A using LASSO; Supplementary Fig. S14 using Elastic Net). With additional removal of variance related to average frame-wise displacement (averaged across sessions), all prediction accuracies were reduced, although overall a similar pattern remained (Supplementary Fig. S15).

**Fig. 4.**
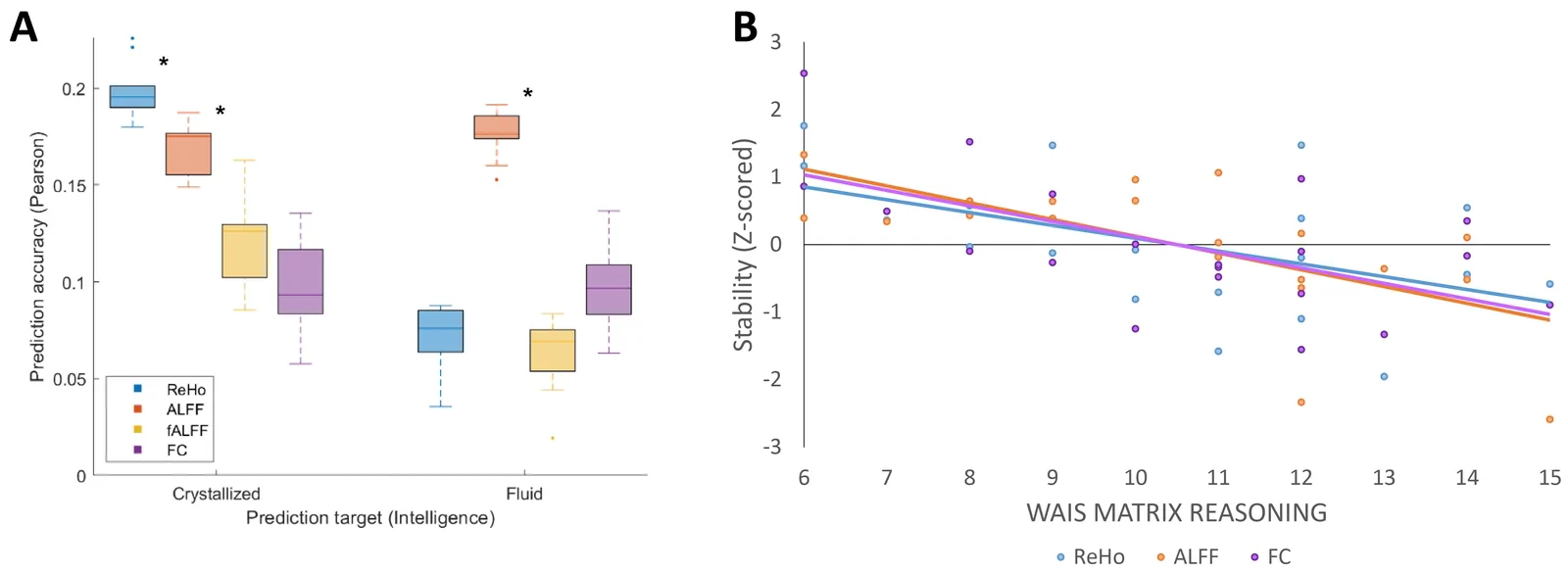
(A) Prediction of crystallized and fluid intelligence using the stability of fingerprints of the nine sessions of the Human Connectome Project sample. Each boxplot shows the prediction performance across five cross-validation runs using LASSO (results using Elastic Net are similar and reported in Supplementary Fig. S14). *Asterisks indicate median cross-validation prediction accuracy (Pearson’s r) > 0.15 across folds. (B) Scatterplot between the WAIS Matrix Reasoning scores and the stability of the task fingerprints in the SZ sample of the OpenNeuro.ds000115 dataset. ReHo = regional homogeneity; ALFF = amplitude of low-frequency fluctuations; fALFF = fractional ALFF; FC = functional connectome.

### 3.6 Local fingerprints are also stable in a clinical sample

In addition to the healthy population, neural fingerprints must be stable within clinical samples to be useful as clinical biomarkers. Studies have shown local abnormalities in schizophrenia (Shukla et al., 2010; P. Wang et al., 2019; Zhao et al., 2019), but whether these abnormalities are stable is unclear. Kaufman et al. (2018) previously showed decreased FC fingerprint stability in patients with schizophrenia by combining three datasets (Kaufmann et al., 2018). Therefore, in two publicly available datasets, we compared the identification accuracy of the fingerprints of patients with healthy controls.

First, we used the publicly available OpenNeuro.ds000115 (Repovš & Barch, 2012) part of this previously reported sample, which includes 20 individuals with schizophrenia (SZ) and 19 healthy controls (see details in Supplementary Materials). fMRI data from the 0- and 2-back tasks obtained on the same day were used. This dataset does not contain rs-fMRI data. ROIs were again created with 3-mm radius spheres around the Power coordinates (Power et al., 2011). Patients and controls were analyzed separately to test whether fingerprints were also stable in a clinical sample with characteristics similar to those of the control sample. Identification accuracies for ReHo and ALFF were 100% for both controls and patients (analyzed separately), while they were lower in SZ for fALFF (0-back to 2-back 60%; 2-back to 0-back: 70%; mean: 65% in SZ; 90% in HC) and FC (0-back to 2-back: 95%; 2-back to 0-back: 100%; mean: 98% in SZ; 100% in HC).

As this dataset does not contain rs-fMRI data, we additionally investigated the publicly available HCP Early Psychosis dataset. This dataset includes 2 resting-state fMRI sessions of 70 individuals with non-affective psychosis and 53 healthy controls (see details in Supplementary Materials). Identification accuracies were 100% for all metrics and both patients and HC (analyzed separately). This suggests that the ReHo and ALFF fingerprints are also stable in patients with psychotic disorders.

Next, we investigated the association between the stability of the fingerprints and intelligence in the schizophrenia sample and their matched healthy controls. Machine learning analysis and investigations of the association between fingerprints and intelligence were not attempted here due to the small sample size. Stability was calculated as the Pearson correlation between the fingerprints of the 0-back and 2-back task sessions (ds000115 sample) or resting-state 1 and 2 sessions (HCP EP sample). In the ds000115 sample, we correlated the stability of the fingerprints with the Wechsler Adult Intelligence Scale (WAIS) Matrix Reasoning (variable WAIS_MATRICS_SCALE) and Vocabulary (variable WAIS_VOCAB_SCALED) subtest scores in both the SZ and HC groups. In the SZ group, the stability of the ReHo, ALFF, and FC fingerprints showed a high negative correlation with the WAIS Matrix Reasoning subtest (all r < -0.50; Fig. 4B). In the HC group, only ALFF stability showed a positive correlation with WAIS Vocabulary subtest scores (r = 0.48) (Supplementary Table S2). In the HCP EP sample, we correlated the stability of the fingerprints with the Fluid Cognition Composite Score (variable nih_fluidcogcomp_unadjusted) and the Crystallized Cognition Composite Score (variable nih_crycogcomp_unadjusted) while controlling for age. No significant correlations were found (see Supplementary Table S3).

### 3.7 Vascular signal is captured with all fingerprints

Altogether, thus far, our findings suggest that ReHo is most stable within an individual and captures brain–behavior associations. However, stable non-neural signals might drive its high individual specificity (i.e., artifacts, breathing, cardiovascular effects). Indeed, earlier studies have shown high motion and cardiac pulsatility stability and reduced test–retest reliability of individual edges within the FC after artifact removal (Noble et al., 2019). Therefore, as the last follow-up analysis, we investigated the contribution of vascular activity to local and global fingerprints. Previous research showed that breath holding is a reliable estimate of the influence of vascular activity on BOLD signals (Yuan et al., 2013), as it induces hypercapnia: an increase in carbon dioxide causes increased cerebral blood flow. This was investigated with the resting-state and breath-holding fMRI sessions (TR of 1400 ms) provided in the enhanced Nathan Kline Institute dataset (eNKI; n = 450; see Supplementary Materials for details) (Nooner et al., 2012), as the HCP does not include breath-holding fMRI data. The breath-holding paradigm consists of alternating breathing in, out, and breath holding, causing a global change in oxygenation. We only used the breath-holding blocks to derive the fingerprints. We observed the highest identification accuracies between resting state and breath holding with ALFF (99%), followed by ReHo (87%), FC (72%), and fALFF (44%). Given that the breath-holding condition induces vascular and hemodynamic changes through hypercapnia (Yuan et al., 2013), the observed identifiability between resting state and breath holding indicates that fingerprinting features are not exclusively driven by neural activity but are also sensitive to vascular-related contributions to the BOLD signal. Identification accuracies for ALFF and ReHo reduced slightly when regressing out variance explained by ReHo and ALFF, respectively (ALFF: from 99% to 98%; ReHo: from 87% to 86%). This result suggests that the vascular signal in resting-state fMRI is captured with all fingerprints, but especially with ALFF. However, these results do not allow for a direct quantification of vascular versus neural contributions; rather, they suggest shared variance between vascular reactivity and individual-specific fingerprint structure.

## 4 Discussion

Here, we compared the properties of three local fingerprints with the commonly reported FC fingerprint, as higher test–retest reliability of these local measures has been shown compared with the FC. Using a publicly available dataset and four different measures of identification performance, different parcellations, and ROI sizes, our findings show high accuracy of local functional fingerprints, especially when using ReHo. Next, we conducted four follow-up analyses. First, we show that the dorsal attention and frontoparietal networks contained the highest number of individual-specific ROIs in the ReHo fingerprint. Second, we demonstrate that intelligence can be predicted with local fingerprints. Moreover, even though the *intraindividual variability* of local fingerprints is small, it *is* meaningful given that it captures interindividual differences in intelligence. Third, using two other publicly available datasets, we show the potential for applying local fingerprints in clinical populations, as the ReHo fingerprint was highly accurate in individuals with a psychotic disorder, and its stability correlated with intelligence in individuals with schizophrenia. As such, our findings suggest the ReHo fingerprint as a good candidate for further exploration: it has excellent fingerprint properties and is predictive of interindividual differences in cognition.

### 4.1 “Fingerprinting” with local fingerprints versus the functional connectome

ReHo quantifies the synchronization among the voxel-wise time series within an ROI, and its stability across sessions appears to reflect the existence of regions that work relatively uniformly across mental states (i.e., tasks). The FC, on the contrary, captures dynamic and state-dependent interactions between regions. Because successful identifiability relies on features that are stable within individuals across time and conditions while being distinctive between individuals, the greater state dependence of the FC may limit the stability of FC-based fingerprints across sessions and, consequently, their suitability for subject identification. Our findings suggest that first-order statistics such as ReHo and (f)ALFF show different patterns of interindividual variation compared with second-order statistics such as the FC (Liégeois et al., 2017). One factor that might play a role is that for ALFF and fALFF, the values are averaged for a region, which reduces noise (T. Xu et al., 2016). ReHo, as implemented in the main analyses of the present study, was computed for each atlas coordinate using the time series of all voxels within a 3-mm sphere, yielding a single value per region reflecting local synchronization within that spatial neighborhood. To our knowledge, no studies have reported identification accuracies for ReHo or (f)ALFF fingerprints thus far. Two studies reported identification accuracies for a different measure of local brain function called BOLD variance (Finn et al., 2015) or BOLD variability (BV) (Gaut et al., 2019). The BV fingerprint reflects the BOLD variance per region, that is, regional fluctuations similar to the ALFF fingerprint but in the full frequency range. These studies showed lower identification accuracies of the BV fingerprint than the FC fingerprint, suggesting that not *all* local fingerprints have better fingerprint properties than the FC and that the BV fingerprint captures processes differently from ALFF.

Similarly, identification performance has not been systematically evaluated for alternative functional connectivity measures such as multivariate distance correlation (Geerligs et al., 2016), the accordance measure (Yoo et al., 2018), or seed-based functional connectivity. These approaches have primarily been used to characterize connectivity patterns or predict behavior rather than for subject identification. A key methodological consideration is that the metrics compared here differ in how regional information is constructed. ReHo provides one value per region based on local synchronization within a 3-mm sphere around each atlas coordinate. ALFF and fALFF are computed at the voxel level and subsequently averaged within each region. In contrast, the FC matrix is obtained by averaging voxel time series within each region and then estimating pair-wise connectivity between regions. Thus, differences in identification performance may reflect not only differences between local and connectivity-based measures, but also differences in how information is aggregated across space and summarized at the regional level. While alternative connectivity formulations such as seed-based functional connectivity or multivariate measures have been proposed in the literature, these approaches were beyond the scope of the present study. We, therefore, focused on the widely used ROI-to-ROI connectivity matrix as the primary representation of functional connectivity.

Examining the forensic identification accuracies, the differences in intraindividual and interindividual variability between the fingerprints become clearer. When fingerprint dissimilarity is used, the FC fingerprint shows near-zero true-positive and false-positive rates, indicating a failure to match any test subjects. This suggests high intra-individual variability and high interindividual variability, that is, a unique but unreliable fingerprint. However, when using the pattern of distances between fingerprint elements, both true-positive and false-positive rates increase significantly, suggesting that while many correct matches occur, so do many incorrect ones. This suggests a decrease in intra-individual and interindividual variability, that is, a more reliable but less unique fingerprint. The pattern of distances between fingerprint elements yields a fingerprint more similar to one another and potentially more noisy, reducing their distinctiveness for identification purposes. Further work is needed to investigate why this pertains especially to the rs–task combinations, compared with the rs–rs combination. Altogether, these results suggest consistency of the ReHo fingerprint across mental states while the FC fingerprint is more variable. Additionally, the high dimensionality of the FC fingerprint may present a challenge for the classifier.

### 4.2 The dorsal attention and frontoparietal networks contribute most to individual “uniqueness”

We investigated brain regions that contribute most to individual uniqueness of the ReHo fingerprint by optimally weighting ReHo features higher in some brain regions to enhance the fingerprint efficacy. Each region was assigned to one of the previously described seven networks based on the mode of the network membership of the voxels within that ROI (Yeo et al., 2011). We found that regions in the dorsal attention and frontoparietal networks contributed the most to identifiability. This suggests that these regions have highest interindividual variability. This is in line with previous studies that predominantly showed the contribution of frontoparietal regions or “higher-order networks” to FC identifiability (Airan et al., 2016; Amico & Goñi, 2018; Cai et al., 2021; Finn et al., 2015; Miranda-Dominguez et al., 2014). For example, one study found that ROIs contributing most to the “uniqueness” of the FC were located in the default mode, attention, and executive control networks (Airan et al., 2016). Previous research has indeed shown greater interindividual variability of multimodal association networks (i.e., default, dorsal attention, and executive control) (Laumann et al., 2015; Mueller et al., 2013; T. Xu et al., 2016), while unimodal networks (i.e., visual and sensorimotor) had lower interindividual variability (T. Xu et al., 2016). Findings on intraindividual variability show an opposite pattern. The DMN, attention, and subcortical systems are relatively less dynamically diverse (Bansal et al., 2019) and thus show lower intraindividual variability, while unimodal networks have higher intraindividual variability (T. Xu et al., 2016). Moreover, it appears that higher-order networks are affected more by genetic and environmental factors (Gao et al., 2014; T. Xu et al., 2016), while lower-order networks are influenced more by situational or task demands (T. Xu et al., 2016).

Our findings also show that regions in the limbic network contributed the least to “uniqueness”. However, some critical considerations should be noted. First, limbic regions in these networks are smaller than ROIs within the other networks and, therefore, might be affected more by normalization errors that induce noise. Second, they are also affected most by magnetic susceptibility artifacts causing a reduced signal-to-noise ratio leading to reduced homogeneity (Aiello et al., 2015), which can make the ReHo fingerprint noisier and hence less specific in these regions. Last, the nature of the fMRI sessions used to investigate uniqueness (the motor task and resting-state sessions) might also influence the contribution of certain ROIs to the uniqueness of the fingerprint (Barch et al., 2013). Altogether, our findings suggest that not only the *distributed connectivity* but also the *local brain function* within higher-order brain networks contributes most to the “uniqueness” of a brain. These networks that are stable within an individual but highly variable across individuals might provide a fundamental backbone of functional brain organization implicated in cognition and disease (Bansal et al., 2019). Future studies might use the localizing power of local fingerprints to attempt to pinpoint “uniqueness” to even smaller regions.

### 4.3 Fingerprints and fingerprint stability capture interindividual differences

Our findings thus far showed that the ReHo fingerprint is an accurate fingerprint. Next, we showed that overall, the local fingerprints are more predictive of crystallized and fluid intelligence than the FC fingerprint. Previous studies significantly predicted fluid intelligence from the FC fingerprint itself (Finn et al., 2015) and the strength of the dynamic FC fingerprint (J. Liu et al., 2018), a high-resolution FC fingerprint (Mansour L et al., 2021), and an FC fingerprint refined using an autoencoder network (Cai et al., 2021). These studies did not attempt to predict crystallized intelligence (i.e., the composite score of the Picture Vocabulary Test and Oral Reading Recognition Test). Only the last mentioned study investigated one crystallized intelligence subtest, the Picture Vocabulary Test—measuring language comprehension—and found that the refined FCs could significantly predict it (Cai et al., 2021). The predictiveness of local fingerprints has not been investigated, although a few studies found significant associations between (voxel-wise) whole-brain ReHo or ALFF and intelligence (C. Li et al., 2018; L. Wang et al., 2011). In short, our findings suggest that different fingerprints will be more appropriate for predicting different traits, just as some fMRI tasks are more appropriate for capturing interindividual differences in specific traits (Greene et al., 2018).

We showed high stability of the local fingerprints. However, it has been shown that reliability is not correlated with predictiveness (Noble et al., 2017) (of FC edges in the referenced study), conceivably because behavior is not static but dynamic (Finn & Rosenberg, 2021). Therefore, we next tested whether the stability of fingerprints across sessions is still informative of individual-level intelligence. Using a machine learning framework, we predicted intelligence with the stability of the fingerprints across sessions. Our results showed that the stability of ReHo could predict crystallized intelligence but not fluid intelligence. However, ALFF stability could predict both crystallized and fluid intelligence, while the stabilities of fALFF and FC fingerprints were not predictive. Thus, even though the ReHo and ALFF fingerprints are highly similar across sessions, the (small) variation due to changes in mental state is still predictive of individual-level cognition.

Our findings suggest that intraregional information processing, as captured with ReHo, is stable across tasks or mental states. This stability of information processing *within* a region is associated more with the more static form of intelligence conceptualized with crystallized intelligence than with the more dynamic ability to reason and solve new problems referred to as fluid intelligence, which might be associated more with brain dynamics or interactions *between* regions. In a previous study in which functional connectivity patterns were also more predictive of crystallized than fluid intelligence, the authors suggested that this might be explained by the robust mapping between brain anatomy and language. In contrast, fluid intelligence might rely on the functioning of multiple (partly) overlapping brain networks involved in many distinct cognitive skills (Dhamala et al., 2021). Our findings show that ALFF was predictive of both crystallized and fluid intelligence. ALFF reflects temporal variability and, therefore, might be more suited for capturing dynamic changes or behaviors. Along this line, a study examining time-varying network configurations found that dynamic configurations are not only good fingerprints in healthy individuals, but that the individual variability in one of the configurations (i.e., brain states) was predictive of florid psychotic symptoms in individuals with a psychotic disorder (Reinen et al., 2018). Future studies could systematically investigate the ability of different fingerprints to predict different types of behavior and cognitive functions.

### 4.4 Local fingerprints are also stable in a clinical sample

The application of biomarkers in precision psychiatry requires stability in clinical populations. Using the OpenNeuro.ds000115 dataset, we show perfect identification accuracies (100%) for ReHo and ALFF in both SZ and HC. For fALFF and FC, identification accuracies for SZ were lower than HC, albeit very slightly for FC. The higher identification accuracies for the FC in this sample than the HCP YA sample (98–100% vs 54–100%) could be due to the smaller sample size (19 SZ and 20 HC vs 399 HC), longer task duration than some (but not all) HCP sessions (±4 minutes vs ±2–14,5 minutes), and the (increased) engagement of similar networks during 2- and 0-back tasks, all of which have been shown to increase FC fingerprint identification accuracy. Systematically testing the effects of sample size and task duration on identification accuracies of the FC falls outside the scope of the current study. With the HCP Early Psychosis dataset, we show perfect identification accuracies (100%) for all fingerprints in both patients with non-affective psychosis and HC. This aligns with our findings in the HCP YA sample in which identification accuracies using two resting-state sessions were also perfect for all fingerprints.

The utility of FC fingerprints has been shown in several clinical populations. A delay in reaching FC fingerprint stability has been reported even in individuals with preclinical signs of mental illness (i.e., increased prodromal symptoms across domains of ADHD, schizophrenia, and depression) (Kaufmann et al., 2017). Moreover, some aspects of FC functioning are disrupted—*and* associated with symptom severity—*across* patients with different psychiatric diagnoses, such as primary psychotic disorder (including individuals diagnosed with schizophrenia, bipolar disorder with psychosis, and schizoaffective disorder), and primary affective disorder (including individuals with depression or bipolar disorder) without psychosis, while other aspects were only disrupted in patients with a psychotic disorder specifically (Baker et al., 2019). Lower stability of the whole-brain FC (between 0- and 2-back fMRI tasks) has also been shown in schizophrenia patients (Kaufmann et al., 2018). Altogether, these previous studies demonstrated the transdiagnostic utility of FC fingerprints. While this was unknown for local fingerprints, our findings suggest the utility of local functional fingerprints in individuals with psychotic disorders. Future studies taking a transdiagnostic approach could inform the stability of different functional fingerprints across individuals with different characteristics and their ability to predict different types of behaviors and symptoms.

Next, we investigated the association between fingerprint stability and intelligence. Given the small sample size, we did not attempt machine learning analysis to examine the fingerprints’ ability to predict intelligence. We found that in individuals diagnosed with schizophrenia from the OpenNeuro.ds000115 dataset, *higher* reconfigurations of ReHo, ALFF, and FC fingerprints were associated with higher scores on the Matrix Reasoning subtest, a measure of abstract problem-solving and inductive reasoning, which is considered a part of fluid intelligence. This finding was opposite to what we observed in healthy individuals of the HCP YA sample, which was also not seen in the matched HC of the OpenNeuro.ds000115 dataset (all r < 0.02–0.1). This finding could suggest that individuals with SZ had difficulties performing the 2-back task and failed to engage the networks required for this task’s additional working memory load (compared with the 0-back task). Indeed, a previous study using the same dataset (including 19 SZ and 10 HC) showed lower 2-back performance in SZ than in HC (0.72 vs. 0.81 for 0-back; 0.64 and 0.80 for 2-back) (Repovš & Barch, 2012). In HC, only ALFF stability was associated with the scores on the Vocabulary subtest.

In contrast to the OpenNeuro.ds000115 dataset, with the HCP Early Psychosis dataset, no significant correlations were found between fingerprint stability and intelligence. This might be explained by several factors that differ between the two datasets: (1) the HCP EP sample consists of individuals in an early phase of psychosis, while the OpenNeuro.ds000115 sample consists of patients with longer illness duration, (2) intelligence was measured differently, and (3) use of resting-state fMRI sessions with a lower variation of fingerprint stability in the HCP EP dataset in contrast to task fMRI sessions in the OpenNeuro.ds000115 dataset (see Supplementary Figs. S16 and S17). Altogether, our findings suggest that even though the ReHo and ALFF fingerprints are highly stable, their subtle variation due to mental states is informative of individual-level cognition in patients with schizophrenia.

### 4.5 Vascular signal is captured with all fingerprints

Higher ReHo, fALFF (Aiello et al., 2015), and ALFF (Tomasi et al., 2013) have been associated with higher glucose metabolism, although the strength of this association varies across the brain. As the fourth follow-up analysis, we investigated the contribution of vascular activity to local and global fingerprints. Identification accuracies with the eNKI breath-holding fMRI task and resting-state data suggest that breathing, or vascular signal, is represented more in resting-state ALFF than in resting-state ReHo. Indeed, previous research has shown that ALFF captures this signal (Golestani et al., 2016; Zuo et al., 2010). Furthermore, our results align with the idea that fALFF, as a normalized ALFF, is affected less by physiological signals from large blood vessels and CSF that affect the entire frequency spectrum. Taken together, our results suggest that the vascular signal plays a role in the identifiability of all fingerprints, especially the ALFF fingerprint. Future studies might look into this further.

### 4.6 Better identification performance of local functional fingerprints is replicable across parcellations and samples and resilient to confounding effects

The performance of the evaluated fingerprints is likely influenced by the analytical pipeline (Z.-Q. Liu et al., 2025). In the present study, we explicitly evaluated the robustness of our findings to several methodological choices, including the parcellation scheme, sample size, confound regression, and feature dimensionality. Nevertheless, other analytical decisions, such as preprocessing, nuisance regression, temporal filtering, ROI signal extraction, and connectivity estimation, may also influence fingerprint estimation and identification performance. Therefore, the relative performance of the different fingerprints should be interpreted within the context of the adopted analysis pipeline, and future work should investigate the robustness of these findings across alternative methodological choices (Z.-Q. Liu et al., 2025).

In our study, specifically, we first evaluated the influence of the parcellation scheme. In addition to the results obtained with ROIs created with 3-mm spheres around the Power coordinates, identification was also evaluated using three other parcellations (i.e., Dosenbach, Shen, and Schaefer with 300 ROIs). While the results with the Dosenbach spheres were similar to the Power spheres, for the Shen and Schaefer parcellations, higher identification accuracies were found for ALFF, followed by ReHo, FC, and fALFF (versus ReHo, ALFF, fALFF, FC with Power, and Dosenbach parcellations). One possible explanation relates to the interaction between the atlas characteristics and the computation of ReHo. Throughout this study, ReHo was estimated within predefined ROIs by calculating Kendall’s coefficient of concordance across the voxel-wise time series within each ROI, rather than using the conventional voxel-wise neighborhood approach. We adopted this ROI-constrained formulation to ensure that ReHo was calculated entirely within ROI boundaries, thereby avoiding contributions from voxels outside the region of interest. Although this approach was applied consistently across all analyses, the Power and Dosenbach atlases consist of relatively small spherical ROIs (respectively, 264 ROIs with 3 mm radius, and 160 ROIs with 5 mm radius), whereas the Shen (268 ROIs) and Schaefer (300 ROIs) atlases comprise substantially larger and more functionally heterogeneous parcels. Consequently, the ROI-based ReHo measure may become less sensitive to fine-grained local synchrony and more dependent on parcel size and internal functional heterogeneity, potentially contributing to reduced identification accuracy and altered ranking of ReHo observed with the Shen and Schaefer atlases. This finding further highlights the importance of spatial resolution and parcellation choice when applying ROI-based ReHo. Similarly, larger ROIs have been associated with smoother signals and a greater proportion of low-frequency fluctuations, which may contribute to the relatively improved performance of fALFF (Zhang et al., 2021). Moreover, larger ROIs reflect more macroscopic organization and are more susceptible to partial volume effects, and averaging within them may diminish local fingerprint characteristics. Although previous work reported lower identification accuracies for FC with larger ROIs (X. Li et al., 2021), our findings suggest that local fingerprint measures may also be influenced by parcellation scale. Next, we tested the effect of sample size, as larger sample sizes are essential for application in precision psychiatry, while they are expected to yield lower identification accuracies (K. Li et al., 2021). Therefore, all comparisons were also conducted with the previously reported sample of 125 subjects (Finn et al., 2015). We found that identification accuracies for the FC decreased the most with increasing sample size, while local fingerprints, especially ReHo, were affected the least. Additionally, our finding of highly accurate local fingerprints was resilient against confounding effects (i.e., age, sex, and intracranial volume), as confound removal resulted in the same pattern of identification accuracies, with ReHo yielding the highest accuracies. Confound removal benefited identification accuracies for FC the most and ALFF the least, suggesting higher FC sensitivity and lower ALFF sensitivity to confounding effects. Last, we investigated the effect of fingerprint size on identification accuracies by creating a PCA FC fingerprint of size similar to the local fingerprints. Identification accuracies between resting-state sessions were similar to the local fingerprints (100%) but poorer than the ReHo fingerprint for rest–task combinations. Differential identifiability was highest for the PCA FC fingerprint between resting-state sessions, but lower than ReHo and ALFF for rest–task combinations. Ranking accuracy of this fingerprint was also lower than for ReHo and ALFF. Altogether, our findings indicate that ReHo is a robust neural fingerprint across several methodological choices evaluated in this study, although the relative performance of all fingerprinting approaches may depend on the analytical pipeline.

### 4.7 Limitations

This study has some limitations. First, the contribution of physiological signals to the BOLD signal remains to be fully understood. While we evaluated the contribution of vascular activity to the fingerprints, other physiological signals may also play a role. Future research should investigate the impact of non-neural signals on identifiability more comprehensively. In this study, we used the FIX-denoised resting-state data from the HCP, which is expected to minimize extraneous signals. Both the FIX-denoised resting-state data and the minimally preprocessed task fMRI data were additionally preprocessed, namely with a bandpass filter of 0.01–0.1 Hz, and regressing out 12 detrended motion parameters (only the minimally preprocessed data), mean time courses of WM, CSF, and global signal. However, we cannot entirely exclude the potential influence of physiological signals. However, notably, previous studies have demonstrated that accurate subject identification primarily relies on neural signals rather than nuisance processes such as physiological processes and head motion (Airan et al., 2016; Gaut et al., 2019; Xifra-Porxas et al., 2021). Second, the extent to which structural information (e.g., brain morphology) and errors in functional-to-anatomical registration influence fingerprint identification accuracy remains unclear (Gaut et al., 2019). The ReHo fingerprint might be more sensitive to this. To account for potential sensitivity to individual differences in functional neuroanatomy, future studies should evaluate fingerprint performance using individualized parcellations. Third, the effect of head motion on the association between fingerprints and intelligence cannot be fully eliminated and may still influence the observed associations. Fourth, we created fingerprints based on local brain function, which is one of many possibilities. Other studies investigated fingerprints based on shape, white matter fiber geometry, voxel-wise diffusion density, or combinations (Kumar et al., 2018). Future studies could also explore a multimodal fingerprint including ReHo. Fifth, we analyzed data that were acquired within a short period (i.e., days), but previous research has shown decreasing identification accuracies with increasing periods (i.e., from 90% within 1 month to 66% within 2–3 years) (Ni et al., 2022). Future work could examine the stability of local and global fingerprints over months or years using longitudinal data. Using data of individuals that are included in multiple datasets would allow for investigation of fingerprint stability across scanners, sequences, preprocessing methods, and timepoints. Sixth, we did not control for differences in scan durations between rs- and task sessions. However, earlier research has shown that identification accuracies between task sessions improved when matching scan duration but were still highly variable across sessions (Finn et al., 2017). Seventh, we checked fingerprint stability in two clinical samples of individuals with psychotic disorders. It is unclear whether our findings generalize to other clinical samples and conditions. Finally, local metrics such as ReHo and (f)ALFF were initially proposed for rs-fMRI data, while we also calculated them with task-based fMRI data. However, these data were treated as resting state, as task timing was ignored. Our findings show that ReHo and (f)ALFF are highly stable across resting state and tasks, suggesting that they appear to reflect intrinsic brain function even when influenced by a task.

### 4.8 Conclusion

Investigating associations between brain function and cognition, behavior, or disease characteristics requires within-individual stable representations of brain function (Tipnis et al., 2020). Recent studies have increasingly stressed the importance of neural representations capturing both intraindividual stability and interindividual differences, as both are desired properties for clinical biomarkers (Finn & Rosenberg, 2021; Mantwill et al., 2021). Here, we show that measures of local brain function—especially ReHo—are better fingerprints than the global FC fingerprint, while they also capture interindividual differences in cognition. The ReHo fingerprint is a good candidate for further exploration of applicability in precision medicine.

## Supplementary Material

Supplementary Material

## Data Availability

The following datasets were used: Human Connectome Project Young Adult S1200 release (https://www.humanconnectome.org/study/hcp-young-adult), OpenNeuro.ds000115 (Repovš & Barch, 2012) (https://openneuro.org/datasets/ds000115/versions/00001), HCP Early Psychosis Release 1.1 (https://www.humanconnectome.org/study/human-connectome-project-for-early-psychosis), and enhanced Nathan Kline Institute dataset (Nooner et al., 2012) (http://fcon_1000.projects.nitrc.org/indi/enhanced/). Code for the main analyses is available on GitHub (https://github.com/juaml/local_fc_fingerprint).
