# Supplementary material for: Spatially localized fMRI metrics as predictive and highly distinct state-independent fingerprints
Source: Imaging Neurosci (Camb). 2026 Nov 6;4:IMAG.a.1444. doi: 10.1162/IMAG.a.1444 (PMC13746340; doi:10.1162/IMAG.a.1444)
Supplement: Supplementary file 1 [file IMAG.a.1444_supp.pdf]

Supplementary Materials

Spatially localized fMRI metrics as predictive and highly distinct state-independent fingerprints

Daouia I. Larabi[a,b,c], Martin Gell[a,d], Enrico Amico[e,f], Simon B. Eickhoff[a,b], Kaustubh R. Patil[a,b]

[a]Institute of Neuroscience and Medicine, Brain & Behaviour (INM-7), Research Centre Jülich, Jülich, Germany

[b]Institute of Systems Neuroscience, Medical Faculty, Heinrich Heine University Düsseldorf, Düsseldorf, Germany

[c]Department of Clinical and Developmental Neuropsychology, University of Groningen, Groningen, the Netherlands

[d]Department of Psychiatry, Psychotherapy and Psychosomatics, Medical Faculty, RWTH Aachen University, Aachen, Germany

[e]School of Mathematics, University of Birmingham, Birmingham, UK

[f]Centre for Human Brain Health, University of Birmingham, Birmingham, UK

Corresponding authors

D.I. Larabi

Department of Clinical and Developmental Neuropsychology, Grote Kruisstraat 2-1, 9712 TS Groningen, the Netherlands

E-mail: d.i.larabi@rug.nl

K.R. Patil

INM-7, Forschungszentrum Jülich, Wilhelm-Johnen-Straße, 52428 Jülich, Germany

E-mail: k.patil@fz-juelich.de

Data

Human Connectome Project Young Adult (HCP-YA) sample

Publicly available data from the Human Connectome Project Young Adult sample was used (https://www.humanconnectome.org/study/hcp-young-adult). The Institutional Review Board at Washington University in St. Louis approved data collection. Analysis of the HCP data received ethical approval from the ethics committee of the medical faculty at the University of Düsseldorf (Study No. 4039). The selection of subjects was performed in R version 3.6.1 (R Core Team 2019). Individuals were included in case of the availability of 1) a T1-weighted scan, 2) two resting-state fMRI scans (rfMRI_REST1 and rfMRI_REST2), 3) data on 58 behavioral variables (Kong et al., 2019) and 4) all fMRI tasks (working memory (tfMRI_WM), gambling (tfMRI_GAMBLING), motor (tfMRI_MOTOR), language (tfMRI_LANGUAGE), social cognition (tfMRI_SOCIAL), relational processing (tfMRI_RELATIONAL) and emotion processing (tfMRI_EMOTION)) (Barch et al., 2013). Subjects with reported issues according to the HCP data release updates were excluded. From this sample, subjects were selected that were unrelated (variable Family_ID) with a similar number of selected males and females (Gender) who were matched on age (Age_in_Yrs), education (SSAGA_Educ), race (Race), and BMI (BMI). This resulted in a sample of 399 subjects consisting of 195 females and 204 males. The mean age, education, and BMI were similar between males and females, namely, 27.83 (sd=3.75) versus 29.68 (sd=3.69), 14.08 (sd=1.75) vs. 15.01 (sd=1.86), and 27.03 (sd=4.38) vs. 26.06 (sd=5.59), respectively. The distribution of races was also similar between males and females: Asian/Nat. Hawaiian/Other

Pacific Is. (11 vs. 13), Black or African Am. (28 vs. 29), White (154 vs. 145), unknown or not reported (4 vs. 2), more than one (7 vs. 6). For all subjects, all available fMRI data (i.e., two resting-state fMRI sessions and seven fMRI tasks) were used for the main analyses. Further details on the tasks and acquisition parameters can be found in Barch et al., 2013 (Barch et al., 2013).

We used the preprocessed data provided by the HCP, which includes artifact removal, motion correction, and registration to standard space (Fischl, 2012; Glasser et al., 2013; Jenkinson et al., 2002, 2012; Woolrich et al., 2001). For the resting-state data, the ICA-FIX noise components removed data were used. ICA-FIX cleaned task-based fMRI data was unavailable. We also used the sample from Finn et al. (Finn et al., 2015) (n=125) for replication and optimization analyses; of this sample, 47 subjects overlapped with the n=399 HCP subsample.

Additional minimal preprocessing was performed assuming no prior knowledge of the data, therefore allowing the use of our preprocessing pipeline in a broad range of situations (i.e., task- or resting-state data, variable scan parameters, etc.). This was done in MATLAB release 2019b (MathWorks) and included regressing out the 12 detrended motion parameters (provided by HCP as Movement_Regressors_dt.txt), as well as the mean time courses of WM, CSF, and global signal, and filtering the time series with a bandpass filter of 0.01-0.1 Hz. Next, the fingerprints were calculated for left-right (LR) and right-left (RL) phase-encoding runs and consequently averaged.

OpenNeuro.ds000115 Working memory in healthy and schizophrenic individuals

We examined the OpenNeuro.ds000115 "Working memory in healthy and schizophrenic individuals" dataset (Repovš & Barch, 2012) (https://openneuro.org/datasets/ds000115/versions/00001). Data collection was approved by the Institutional Review Board at Washington University. Analysis of OpenNeuro datasets received ethical approval from the ethics committee of the medical faculty at the University of Düsseldorf (2018-317-RetroDEuA). Subjects were included when data was available of the 0- and 2-back fMRI tasks and intelligence scores. This resulted in the inclusion of 20 patients with schizophrenia (age: mean = 23.7, SD = 3.7; sex: 6 females, 14 males; ethnicity: 8 African American, 12 white) and 19 healthy controls (HC; age: mean = 20.8, SD = 5.2; sex: 8 females, 11 males; ethnicity: 11 African American, 8 white). The fMRI data of the 0-back and 2-back tasks were used for analyses. The WAIS Matrix Reasoning (WAIS_MATRICS_SCALE) and Vocabulary subtest scores (WAIS_VOCAB_SCALED) were used for investigating the correlations between fingerprint stability and intelligence.

Human Connectome Project Early Psychosis sample

We examined data from the HCP Early Psychosis Release 1.1 (https://www.humanconnectome.org/study/human-connectome-project-for-early-psychosis) which includes minimally preprocessed structural data and unpreprocessed fMRI data of 161 subjects. All procedures have been approved by the Partners Healthcare Human Research Committee/IRB. Analysis of the HCP data

received ethical approval from the ethics committee of the medical faculty at the University of Düsseldorf (Study No. 4039). This dataset includes individuals within the first five years of the onset of psychotic symptoms. Given that the subgroup of patients with affective psychosis only included 23 subjects, we focused on the individuals with non-affective psychosis and healthy controls. Furthermore, we selected subjects with data available for 1) all four resting-state scans, 2) fluid and crystallized cognition composite scores, and 3) age and sex. This resulted in the inclusion of 53 healthy controls (age: mean = 25.08, SD = 4.1; sex: 19 females, 34 males; race: African American = 5, Asian = 8, white = 37, more than one race = 1, unknown or not reported = 2) and 70 patients with non-affective psychosis, diagnosed with schizophrenia, schizophreniform disorder, schizoaffective disorder, psychosis NOS, delusional disorder, or brief psychotic disorder (age: mean = 22.15, SD = 3.11; sex: 22 females, 48 males; race: African American = 36, American Indian/Alaska = 1, Asian = 4, white = 26, unknown or not reported = 3).

Enhanced Nathan Kline Institute (eNKI) sample

Data of 450 clinically healthy subjects (i.e., without a psychiatric or neurological diagnosis or use of medication for the treatment of any such conditions) from the community-based sample of the enhanced Nathan Kline Institute dataset were included (mean age = 44.81, SD = 17.5; sex: 304 females, 142 males, 4 unknown or not reported; race: American Indian or Native Alaskan = 3, Asian = 19, Black or African American = 67, Native Hawaiian or Other Pacific Islander = 1, White = 352, Other: 8) (Nooner et al., 2012) (http://fcon_1000.projects.nitrc.org/indi/enhanced/). This subset was selected based on the availability of both resting-state and breath-holding scans, with the exclusion

of subjects with excessive movement or who fell asleep during the resting-state scanning. This study was approved by the Nathan Kline Institute for Psychiatric Research Institutional Review Board. Analysis of the data from eNKI received ethical approval from the ethics committee of the medical faculty at the University of Düsseldorf (Study No. 4039).

Methods

Figures

All figures of identification accuracies were created with R version 3.6.1 (R Core Team 2019) using packages reshape2 (Wickham, 2007), ggplot2 (Wickham, 2016), extrafont (Chang, 2014), and grid.

Effect of sphere size around Power coordinates on identification accuracies - smaller regions of interest (ROIs) yield higher identification accuracies

The effect of sphere size on identification accuracies was investigated, as sphere size is known to affect the calculation of regional homogeneity (ReHo) (Zang et al., 2004). We tested spheres with radiuses of 3, 5, and 10 mm. Using single voxels as ROIs for all measures was not pursued, as this would yield an extremely high-dimensional feature space, making computation impractical and reducing signal-to-noise ratio compared to standard ROI-based averaging.

Replication and additional analyses sample Finn et al. (2015)

To replicate the results shown by Finn et al. (2015), the same sample was included (Finn et al., 2015). One subject was not included as their data was not available (anymore), leaving 125 subjects for analyses. Of these 125 subjects, 47 subjects overlapped with our large HCP sample (n=399). Data from the minimally preprocessed Human Connectome Project (HCP) pipeline of the same sessions reported by Finn et al. (2015) was included. For the resting-state fMRI data, this did not include the removal of noise components as obtained with ICA FIX. Additional preprocessing and calculation of connectivity matrices were done in Matlab 2019b (Mathworks Inc, Natick, MA). This included the removal of 12 motion parameters (provided by HCP as Movement_Regressors_dt.txt files), regression of the mean time courses of WM, CSF, and global signal, removal of the polynomial trend up to order 3, and low pass filtering (FFT; 0-0.26 Hz). No smoothing was applied. The brain was parcellated into 268 regions according to the Shen parcellation (Shen et al., 2013). Pearson correlation coefficients were calculated between the mean time courses of each pair of regions, resulting in 268 × 268 symmetric FC matrices for each session for each subject. This was done separately for the LR- and RL-phase encoding runs and the matrices were consequently averaged. Each element in the resulting matrices (i.e. 125 subjects x 6 sessions) reflects the connectivity between two regions. Next, identification accuracy was calculated. In addition to the FC analyses described in Finn et al. (2015), we also calculated identification accuracies for ReHo, ALFF, and fALFF, using the same sample and preprocessing parameters. For comparison, we also calculated identification accuracies for FC, ReHo, ALFF, and fALFF with three other parcellations (i.e. Power (3-mm spheres), Dosenbach, and Schaefer).

Contribution of regions to individual uniqueness via margin optimization

The two optimization problems RS1-RS2 and RS2-Motor showed similar results with significantly increased margin (Fig. S1).

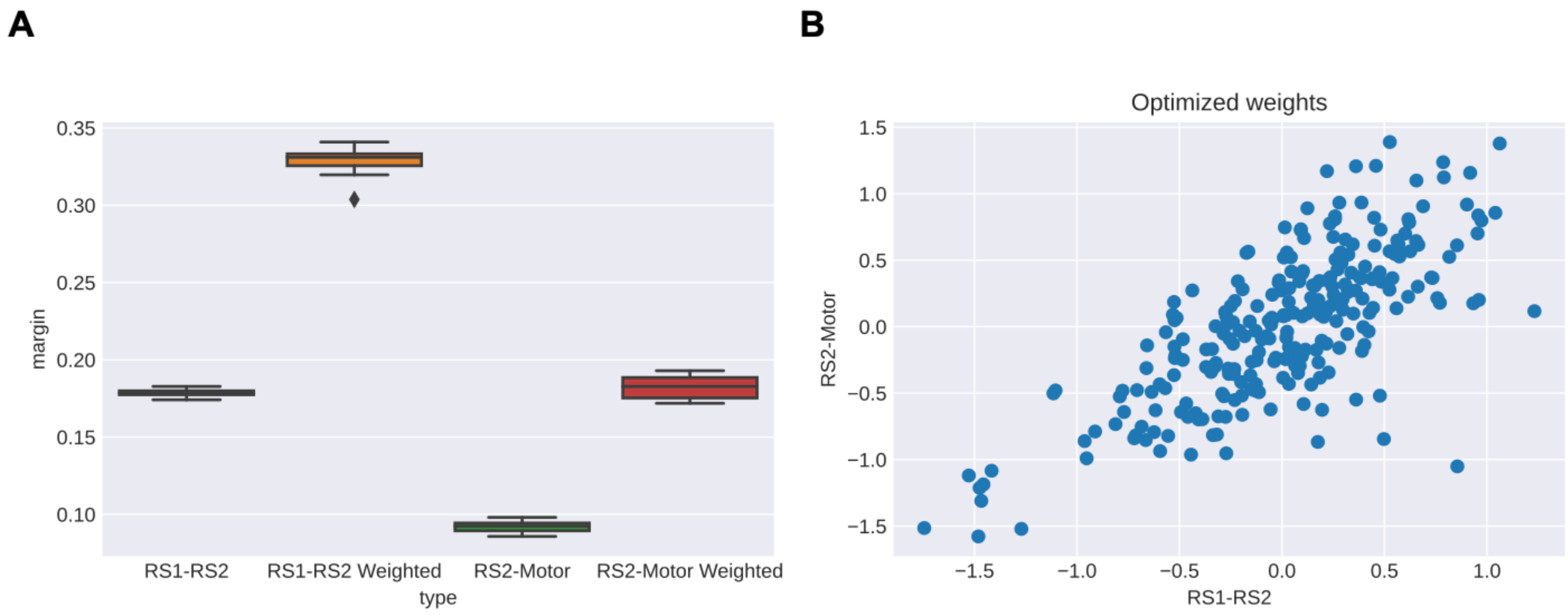

Fig. S1: A) The margin on the 50% out-of-sample subjects improved when using the weights derived from 50% of the data. The procedure was repeated 100 times with random sampling of train-test splits. The margin increased substantially. B) The averaged weights for the two optimization problems (RS1-RS2 and RS2-Motor) were highly correlated.

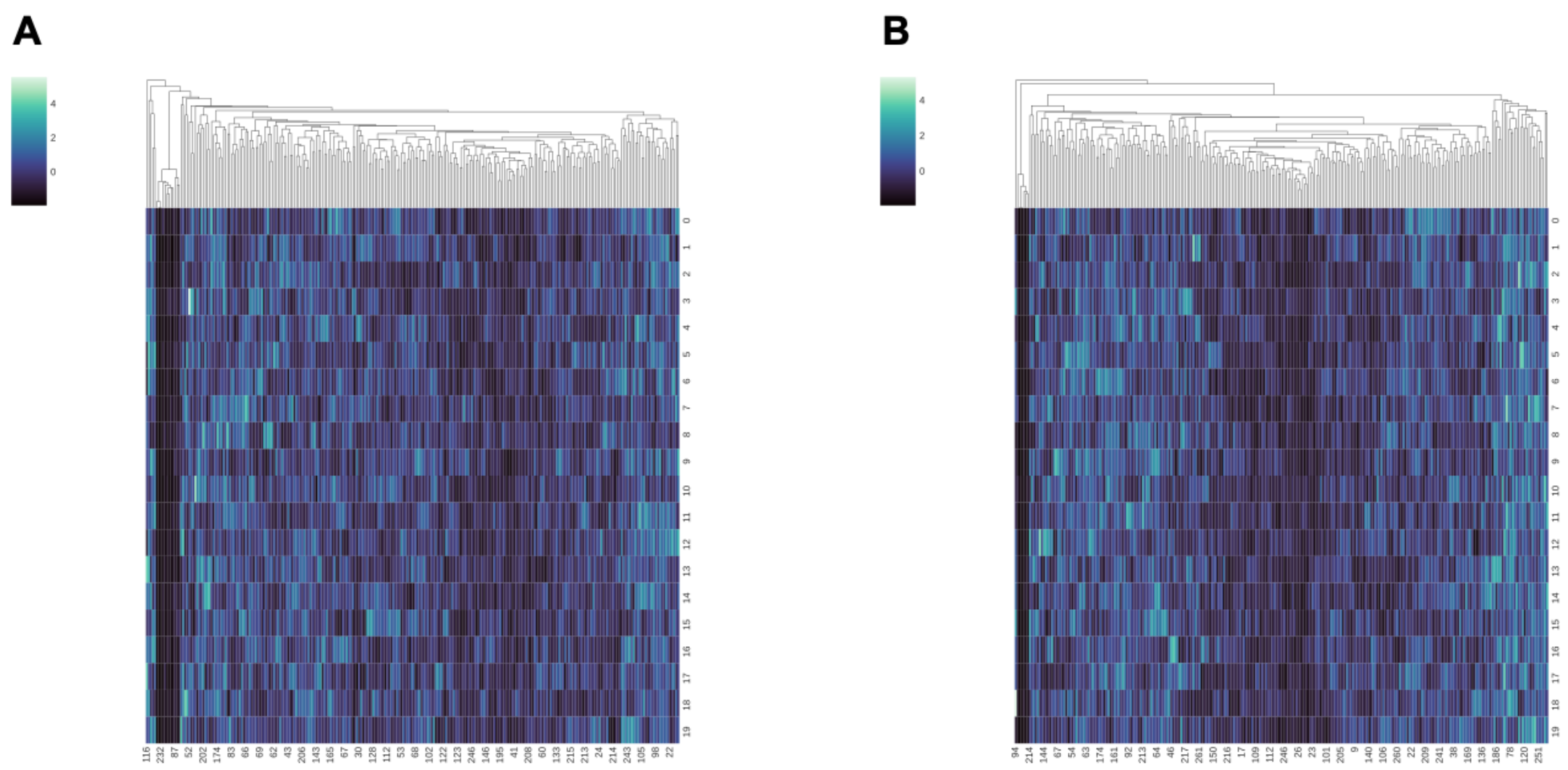

Fig. S2: Weights across 100 runs of the optimization algorithm. The rows are the runs and the columns are the ROIs. A) RS1-RS2 runs. B) RS2-Motor runs. The weights were stable across the 100 optimization runs, therefore, for further analyses, we used the average of the weights across the 100 runs.

Table S1: Proportion of the Power 3-mm ROI assignment to seven Yeo networks.

Network	None	Visual	Somatomotor	Dorsal Attention	Ventral Attention	Limbic	Frontoparietal	Default Mode
Proportion	0.1	0.14	.17	0.1	0.09	0.04	0.13	0.23

Local fingerprints in two clinical samples

We examined the OpenNeuro.ds000115 Working memory in healthy and schizophrenic individuals' dataset. The sample is described in the Data section. The data were preprocessed with fMRIPrep version 1.5.9, which has been described in detail elsewhere (Esteban et al., 2019). Briefly, for the structural MRI images, this included intensity non-uniformity (INU) correction, skull-stripping of the T1w image, followed by tissue segmentation, and spatial normalization to MNI-space using nonlinear registration. For the resting state and breath-holding data, a reference image and its skull-stripped version were first generated. Next, head motion relative to the reference image was estimated and slice-time correction was performed. The BOLD images were then coregistered to the native space of the subjects' T1w image, resampled to correct for motion, and mapped to MNI space. Note that ICA-AROMA denoising was not used as this smooths the data resulting in more spatial homogeneity and consequently more uniform and non-specific ReHo estimates.

Last, mean cerebrospinal fluid and white matter time courses, global signal as well as 12 motion parameters, were regressed out and the data were bandpass filtered (0.01-0.1Hz). Regions were created with 3-mm spheres around the Power coordinates. Identification accuracy was calculated as described in the Methods section of the main text.

Additionally, we examined the HCP Early Psychosis dataset. The sample is described in the Data section. We used the HCP pipelines to minimally preprocess this data (Glasser et al., 2013) (script GenericfMRIVolumeProcessingPipeline) and apply multi-run ICA-FIX cleaning (script IcaFixProcessing). The volumetric time series of four resting state scans (resting state 1 and 2 both acquired with AP and PA phase encoding) were used for analyses. Mean CSF and WM time courses, global signal as well as 12 motion parameters, were regressed out and the data were bandpass filtered (0.01-0.1Hz). Regions were created with 3-mm spheres around the Power coordinates. Next, the fingerprints were calculated for both AP and PA phase-encoding runs and consequently averaged. Identification accuracy was calculated as described in the Methods section of the main text. As the number of patients was higher than the number of healthy controls, we randomly selected a subsample of 53 patients to calculate identification accuracies and the correlations between fingerprint stability and intelligence. This was repeated 100 times and the mean across repetitions was used. The fluid and crystallized intelligence composite scores (nih_fluidcogcomp_unadjusted and nih_crycogcomp_unadjusted; age-adjusted intelligence scores were missing for many subjects) were used for investigations of the partial correlation with intelligence controlling for age.

Signals underlying regional homogeneity

The selection of data of the Enhanced Nathan Kline Institute (eNKI) sample is described in the Data section. Next, the data were preprocessed with fMRIPrep version 20.1.1 which has been described in detail elsewhere (Esteban et al., 2019). Briefly, for the structural MRI images, this included intensity non-uniformity (INU) correction, skull-stripping of the T1w image, followed by tissue segmentation, and spatial normalization to MNI-space using nonlinear registration. For the resting state and breath-holding data, a reference image and its skull-stripped version were first generated. Next, head motion relative to the reference image was estimated and slice-time correction was performed. The BOLD images were then coregistered to the native space of the subjects' T1w image, resampled to correct for motion, and mapped to MNI space. Note that ICA-AROMA denoising was not used as this smooths the data resulting in more spatial homogeneity and consequently more uniform and non-specific ReHo estimates. Lastly, mean cerebrospinal fluid and white matter time courses, global signal as well as 12 motion parameters, were regressed out and the data were bandpass filtered (0.01-0.1Hz). For the breath-holding task, fingerprints were calculated only including the breath-holding (BH) parts of the task (i.e. start 18 seconds after task onset, lasting for 18 seconds, alternating with 18 seconds of breathing in and out). Regions were created with 3-mm spheres around the Power coordinates. Identification accuracy was calculated as described in the Methods section of the main text.

Results

Effect of sphere size around Power coordinates on identification accuracies - smaller ROIs yield higher identification accuracy

We found that sphere sizes of 3 mm yielded the highest identification accuracy for all the fingerprints (Fig. S3). Therefore, the main results are reported with spheres of this size.

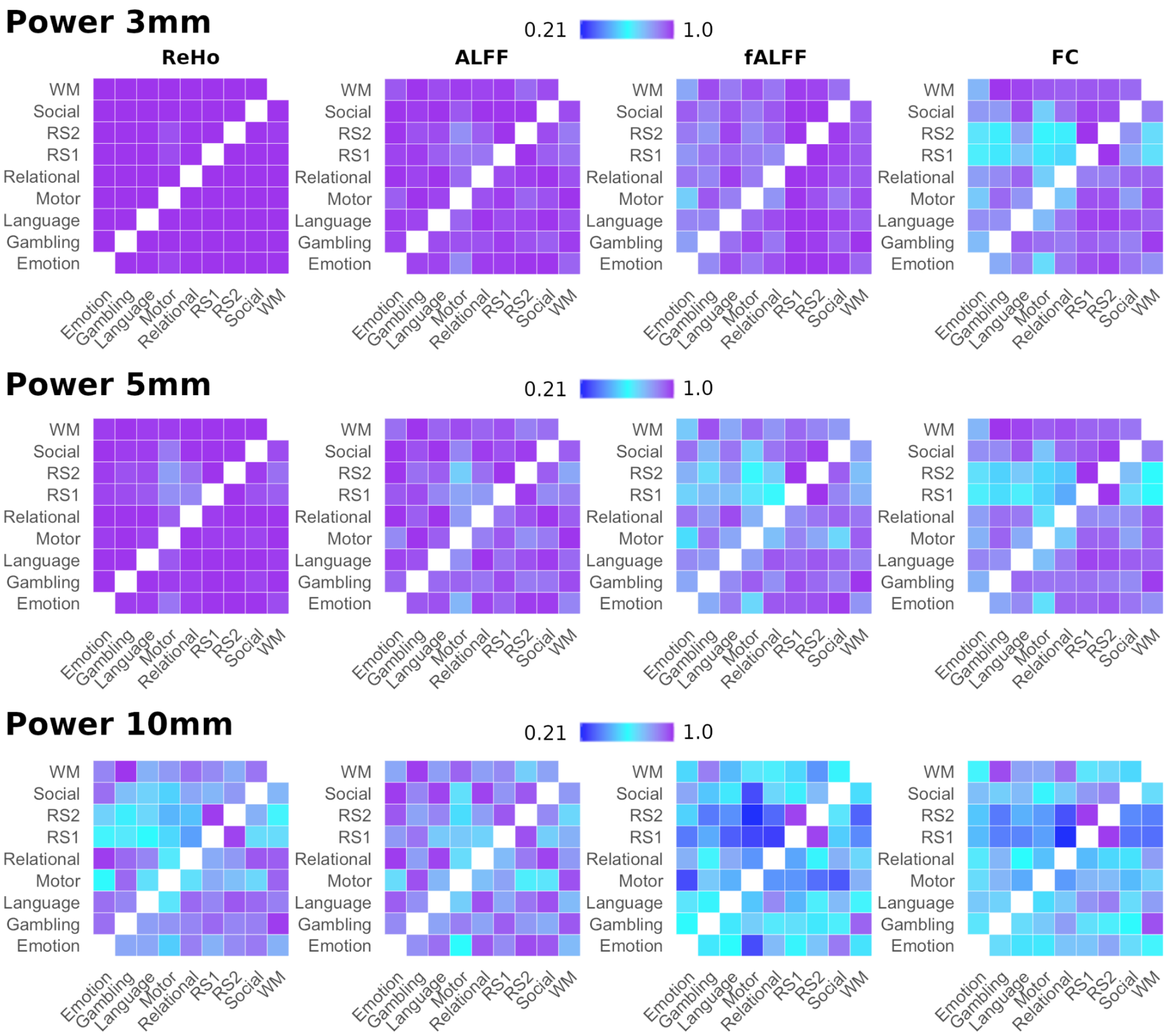

Fig. S3: comparison of identification accuracies for several functional fingerprints with different sphere sizes around the Power coordinates in the Human Connectome Project sample. The highest identification accuracies were seen for 3-mm sphere sizes, followed by 5-mm and 10-mm. Therefore, the 3-mm spheres were used for the main analyses. Abbreviations: ReHo = regional homogeneity; ALFF = Amplitude of Low Frequency Fluctuations; fALFF = fractional Amplitude of Low Frequency Fluctuations; FC = functional connectome; RS = resting state.

Idiff

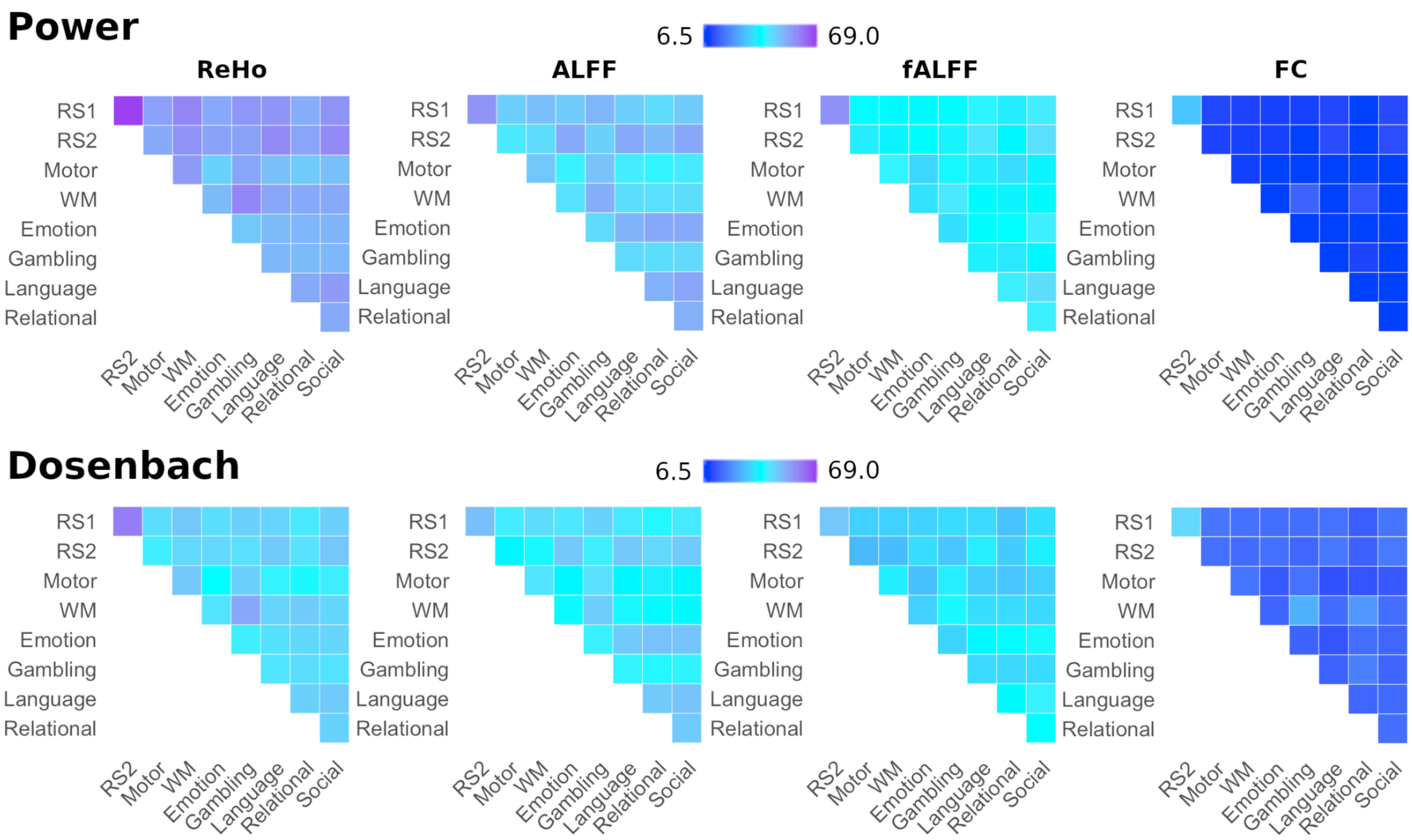

Fig. S4: comparison of Idiff of functional fingerprints in the Human Connectome Project sample. Abbreviations: ReHo = regional homogeneity; ALFF = Amplitude of Low Frequency Fluctuations; fALFF = fractional Amplitude of Low Frequency Fluctuations; FC = functional connectome; RS = resting state.

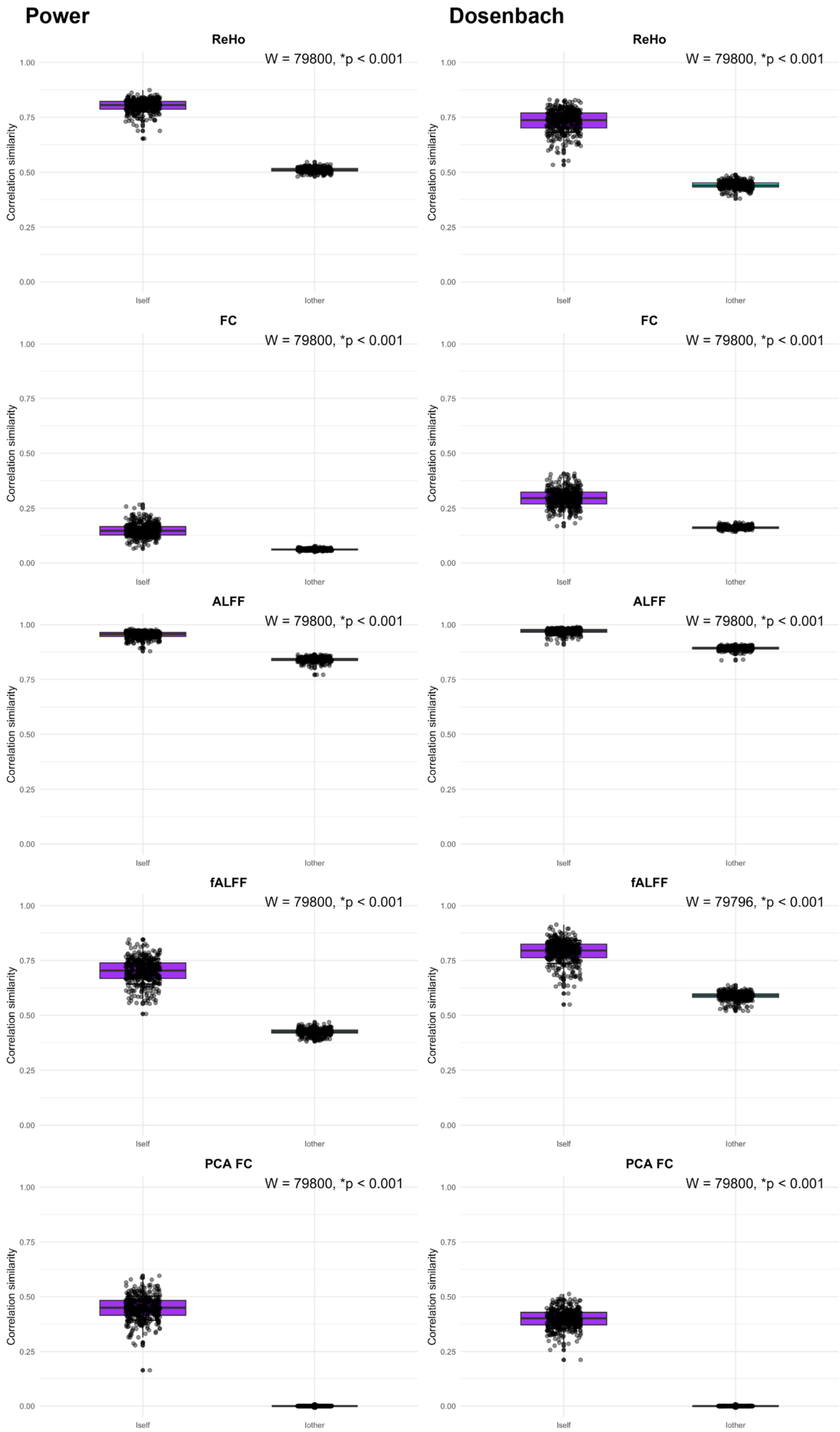
Power
Dosenbach
ReHo
W = 79800, *p < 0.001
FC
W = 79800, *p < 0.001
ALFF
W = 79800, *p < 0.001
fALFF
W = 79800, *p < 0.001
W = 79796, *p < 0.001
PCA FC
W = 79800, *p < 0.001
Correlation similarity
Iself
Iother
0.00
0.25
0.50
0.75
1.00

4

Fig. S5: comparison of Iself (left in each plot) and Iothers (right in each plot) of functional fingerprints in the Human Connectome Project sample. The difference between Iself and Iothers was compared statistically using paired Wilcoxon signed-rank tests. Abbreviations: ReHo = regional homogeneity; ALFF = Amplitude of Low Frequency Fluctuations; fALFF = fractional Amplitude of Low Frequency Fluctuations; FC = functional connectome; RS = resting state.

A similar size FC fingerprint

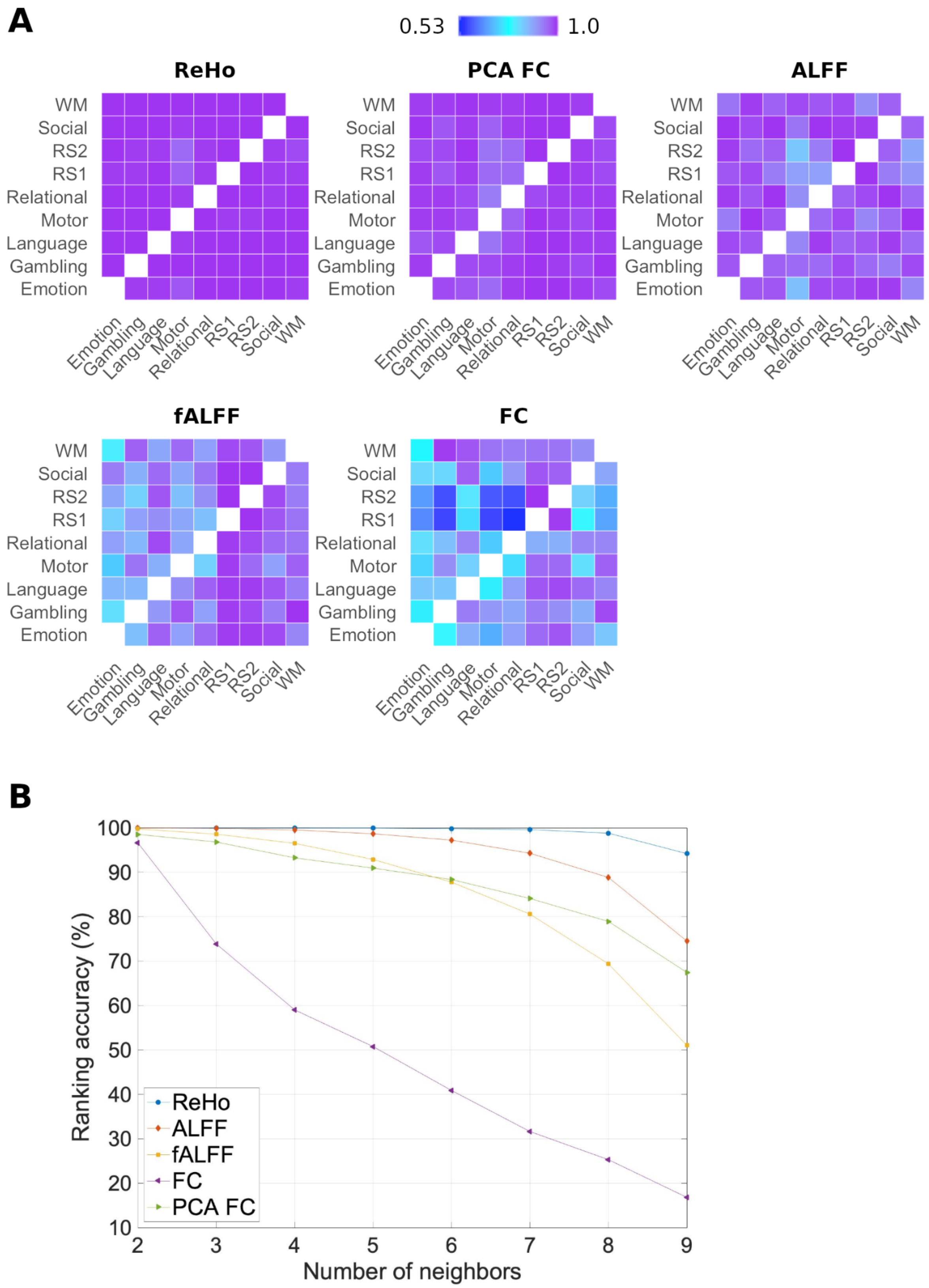

Fig. S6: comparison of identification accuracies (A), and ranking accuracy (B) of the PCA FC fingerprint with other fingerprints in the HCP Young Adult sample.

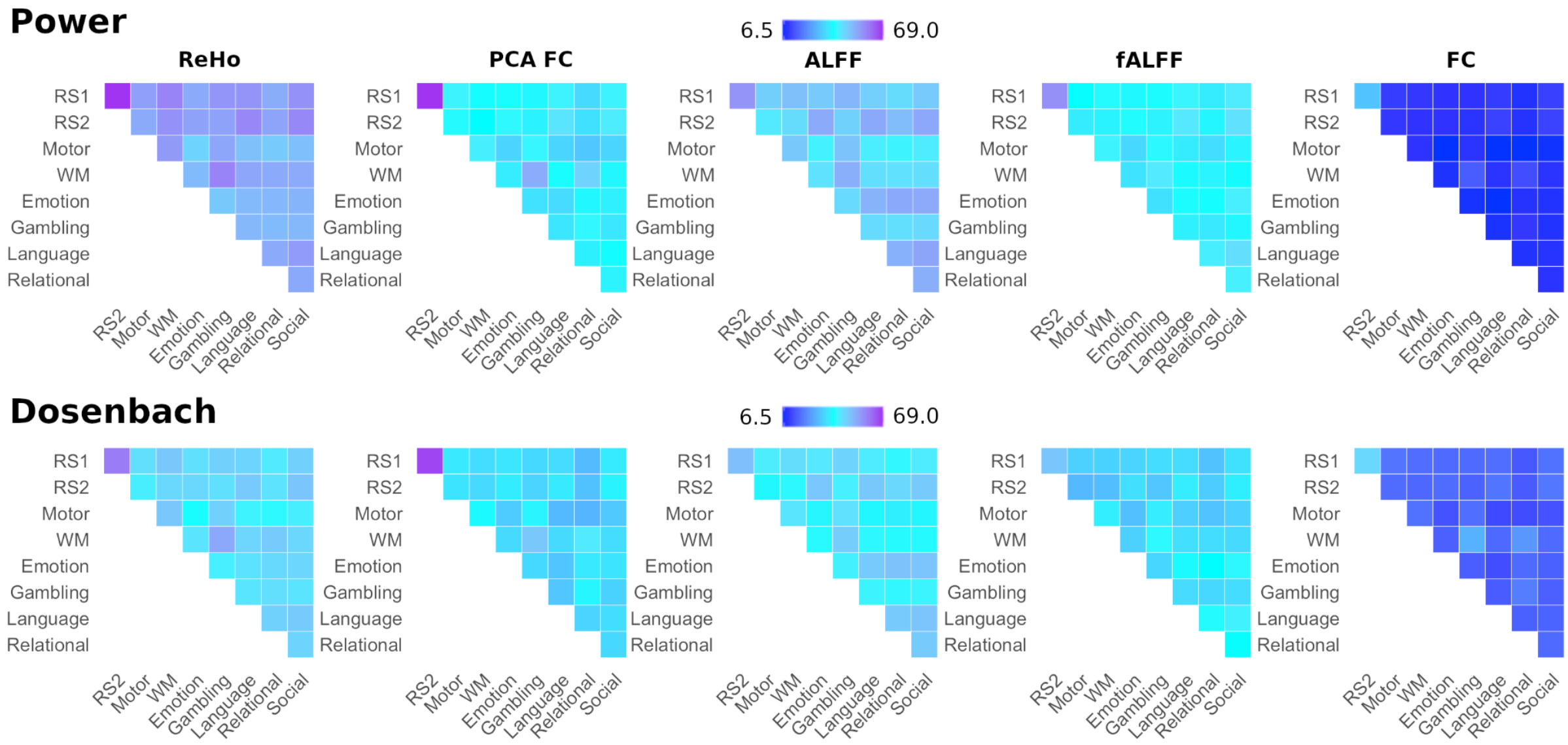

Fig. S7: comparison of Idiff of PCA FC fingerprint with other fingerprints in the Human Connectome Project sample. Abbreviations: ReHo = regional homogeneity; ALFF = Amplitude of Low Frequency Fluctuations; fALFF = fractional Amplitude of Low Frequency Fluctuations; FC = functional connectome; RS = resting state.

Fingerprint identification accuracies with Dosenbach, Shen, and Schaefer parcellations

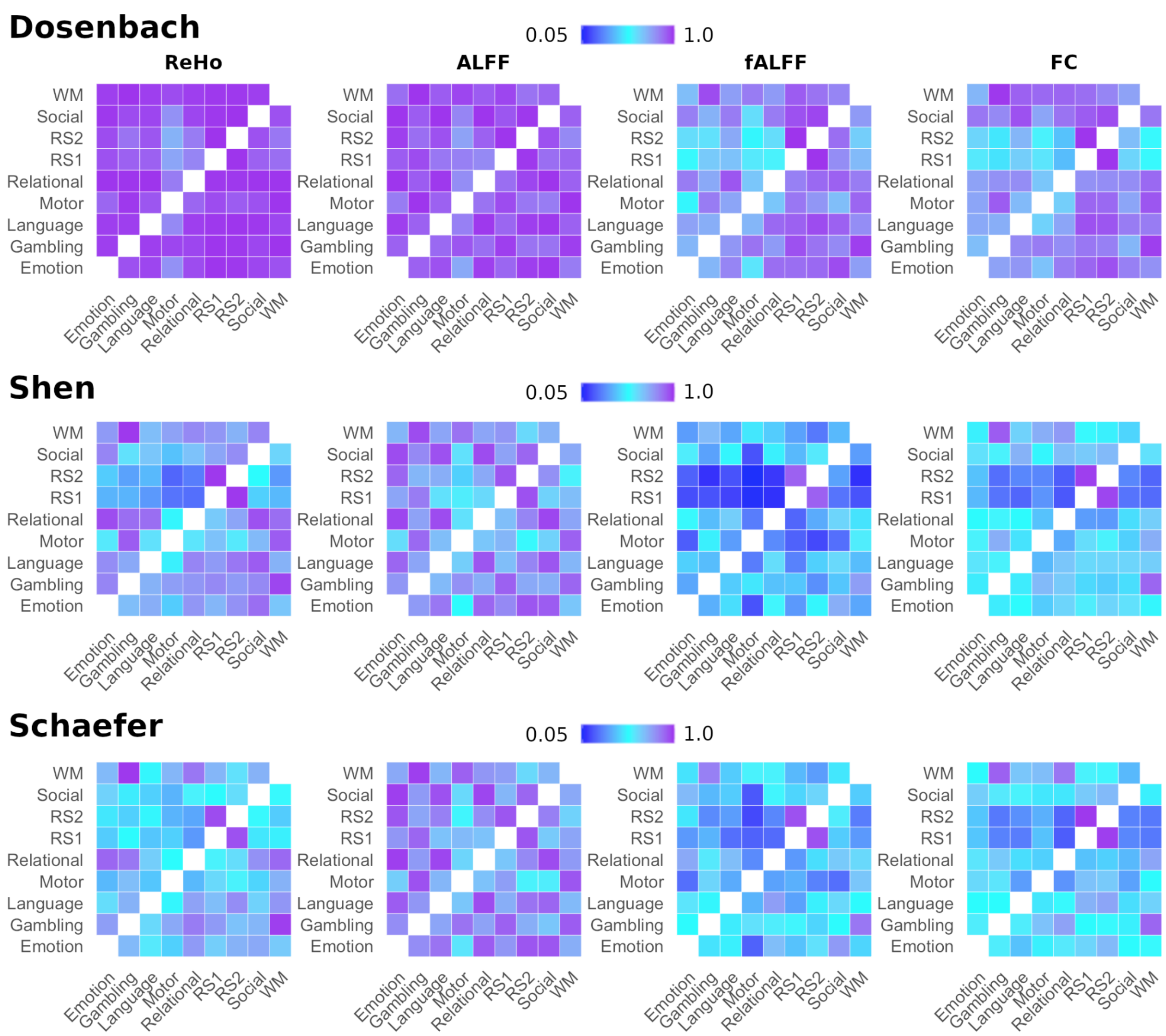

Fig. S8: comparison of identification accuracies of local and global functional fingerprints in the Human Connectome Project sample for the Dosenbach, Shen, and Schaefer 300 parcellations. Abbreviations: ReHo = regional homogeneity; ALFF = Amplitude of Low Frequency Fluctuations; fALFF = fractional Amplitude of Low Frequency Fluctuations; FC = functional connectome; RS = resting state.

Identification accuracies of functional fingerprints in the HCP sample after confound removal

After confound removal, identification accuracies were still highest for ReHo (Fig. S9). In contrast with the results without confound regression, this was followed by FC, fALFF, and ALFF (in contrast to ReHo-ALFF-fALFF-FC). These results suggest a larger influence of confounders on global fingerprints such as the FC.

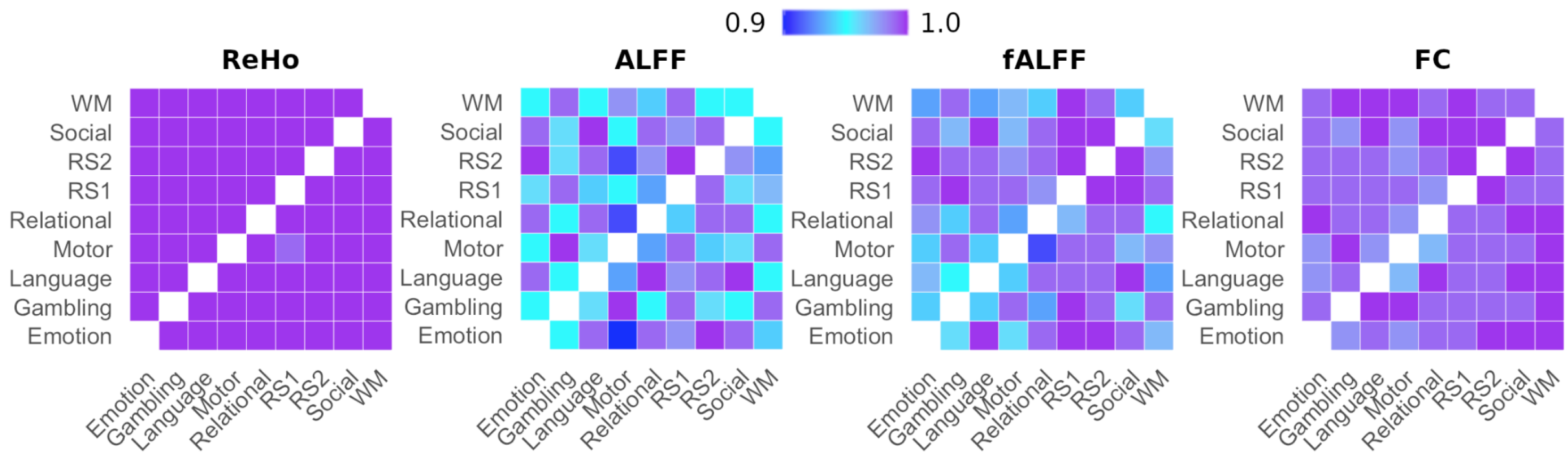

Fig. S9: comparison of identification accuracies of functional fingerprints in the Human Connectome Project sample after confound removal (i.e. sex, age, and intracranial volume). Abbreviations: ReHo = regional homogeneity; ALFF = Amplitude of Low Frequency Fluctuations; fALFF = fractional Amplitude of Low Frequency Fluctuations; FC = functional connectome; RS = resting state.

Replication and additional analyses sample Finn et al. (2015)

First, we replicated the results reported by Finn et al. (2015) (Fig. S10). Finn et al. (2015) reported a mean identification accuracy of 94.4% between the FCs of two resting-state fMRI sessions. We show a mean accuracy of 90% (Fig. S10), which is in line with another replication (91%) (Venkatesh et al., 2020). Accuracies between FCs of task-based fMRI sessions and between resting-state and task-based fMRI

sessions were also similar (i.e. 51-80%) to results reported in Finn et al. (2015) (i.e. > 54%).

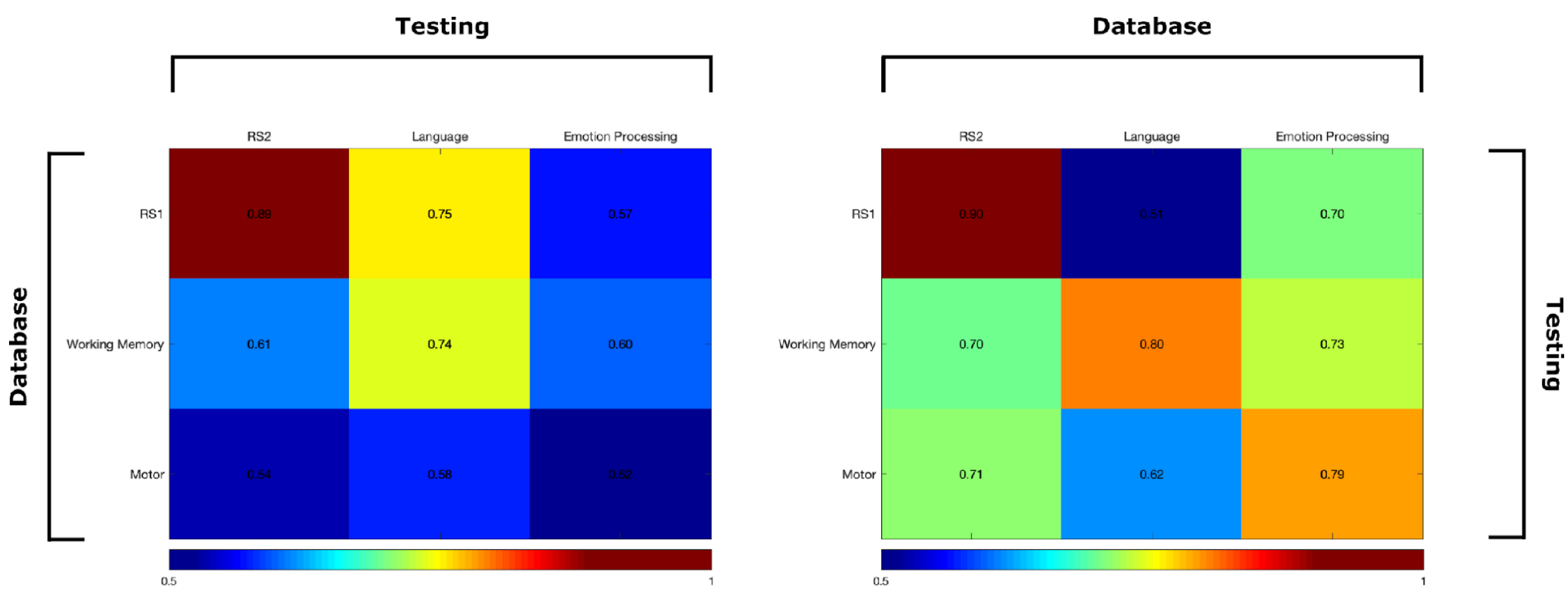

Fig. S10: comparison of identification accuracies of functional connectome (FC) fingerprints based on Pearson correlations between time courses for 125 subjects of the Human Connectome Project sample (replication of (Finn et al., 2015)). Abbreviations: RS = resting state.

Second, we calculated identification accuracies for the FC, as well as the local fingerprints ReHo, ALFF, and fALFF using four different parcellations (Fig. S11). For the Power spheres (3 mm) and Dosenbach parcellation, similarly to the results in the large HCP sample, we found near-perfect identification accuracies for ReHo, followed by ALFF, the FC, and fALFF. One difference was observed compared to results in the large HCP sample: identification accuracy was higher for the FC than for fALFF. Results for the Schaefer parcellation were the same as in the large HCP sample: highest identification accuracies for ALFF, followed by ReHo, FC, and fALFF. Results for the Shen parcellation were slightly different compared to the large

HCP-sample: highest identification accuracies for ReHo, followed by ALFF, FC, and fALFF (versus ALFF, ReHo, FC, fALFF in the large HCP-sample).

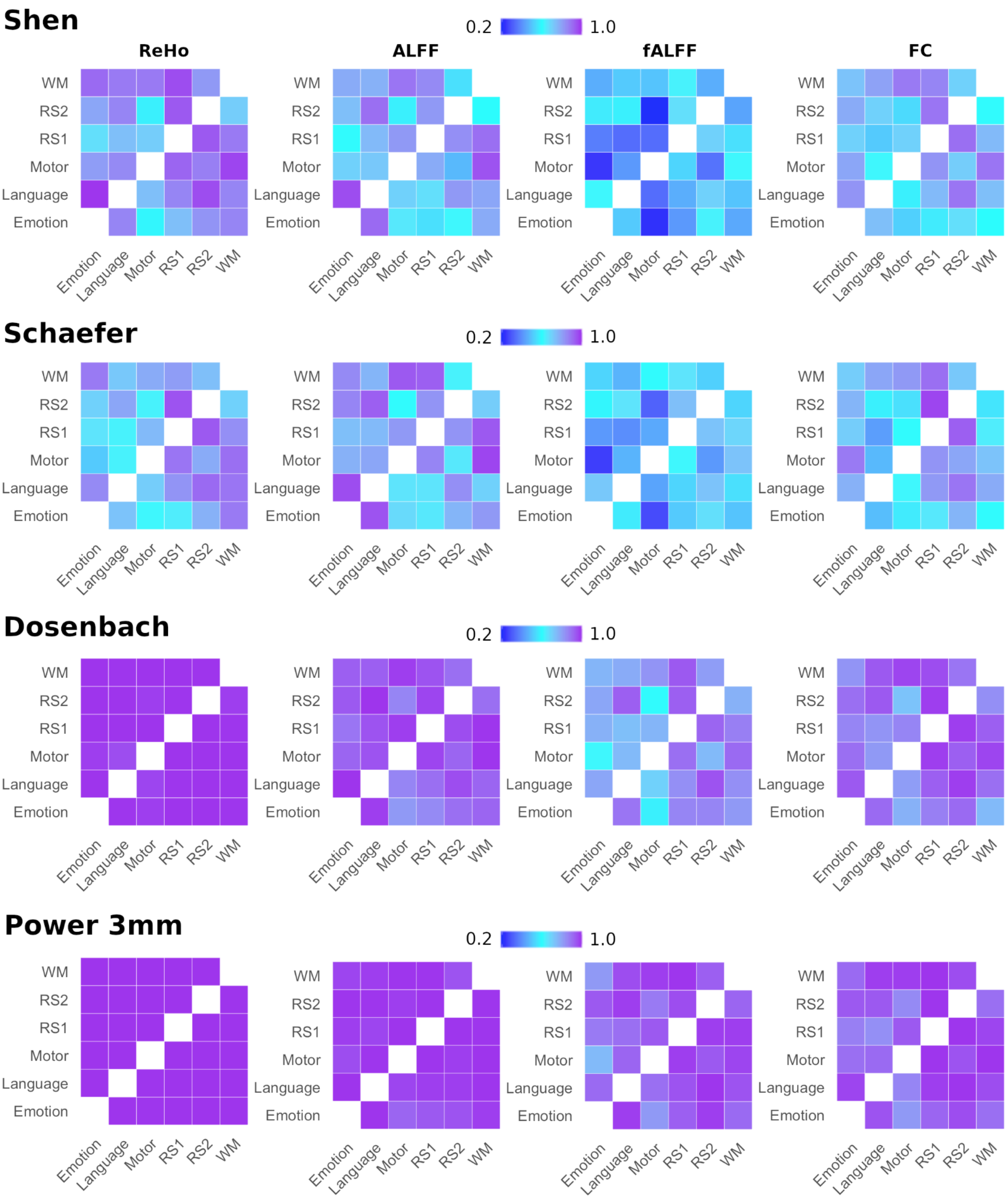

Fig. S11: comparison of identification accuracies of the FC with other functional fingerprints for 125 subjects of the Human Connectome Project sample. The same sample was included and similar preprocessing parameters were used as reported in Finn et al. (2015). Abbreviations: ReHo = regional homogeneity; ALFF = Amplitude

of Low Frequency Fluctuations; fALFF = fractional Amplitude of Low Frequency Fluctuations; FC = functional connectome; RS = resting state.

Fingerprints and fingerprint stability capture interindividual differences

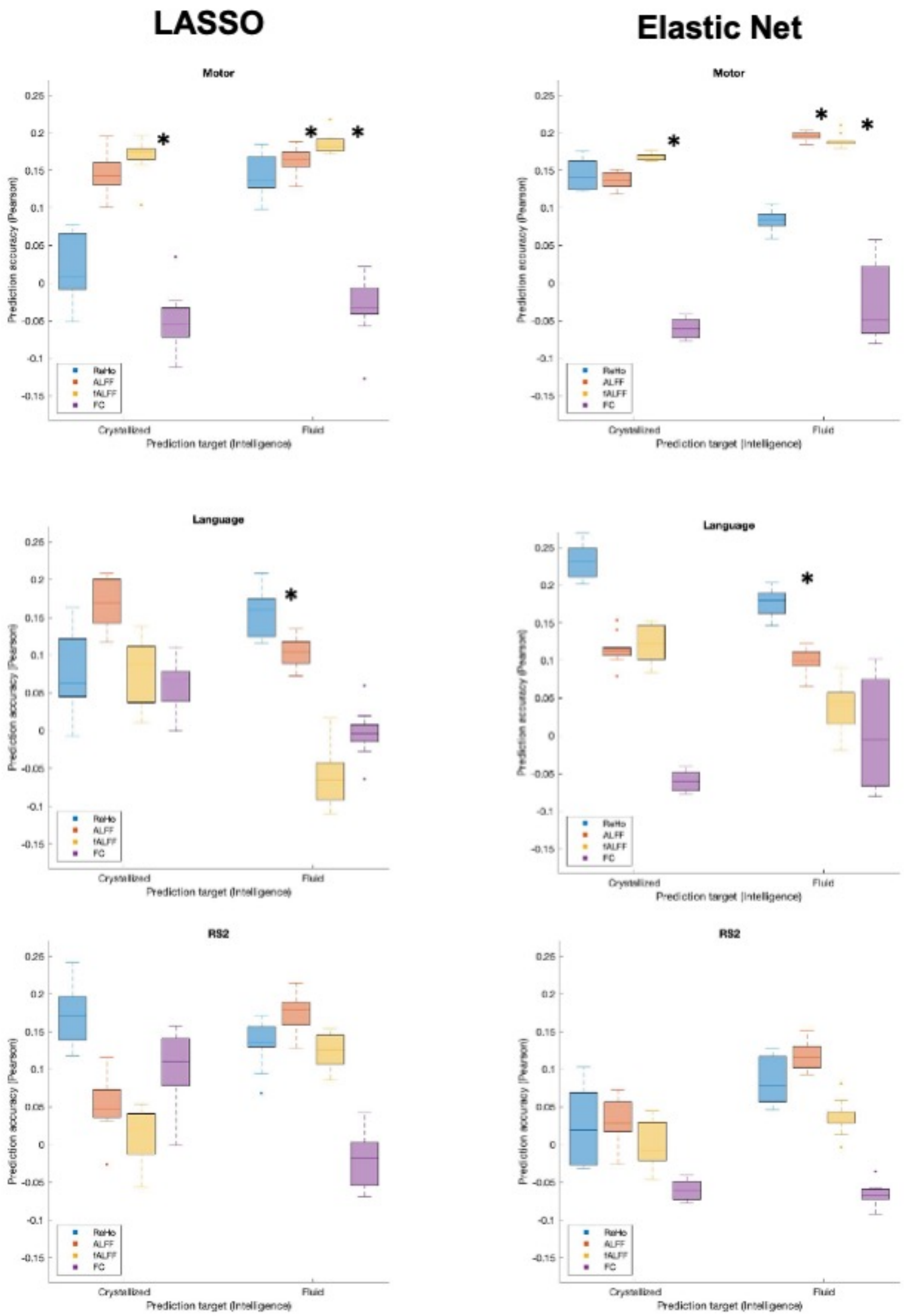

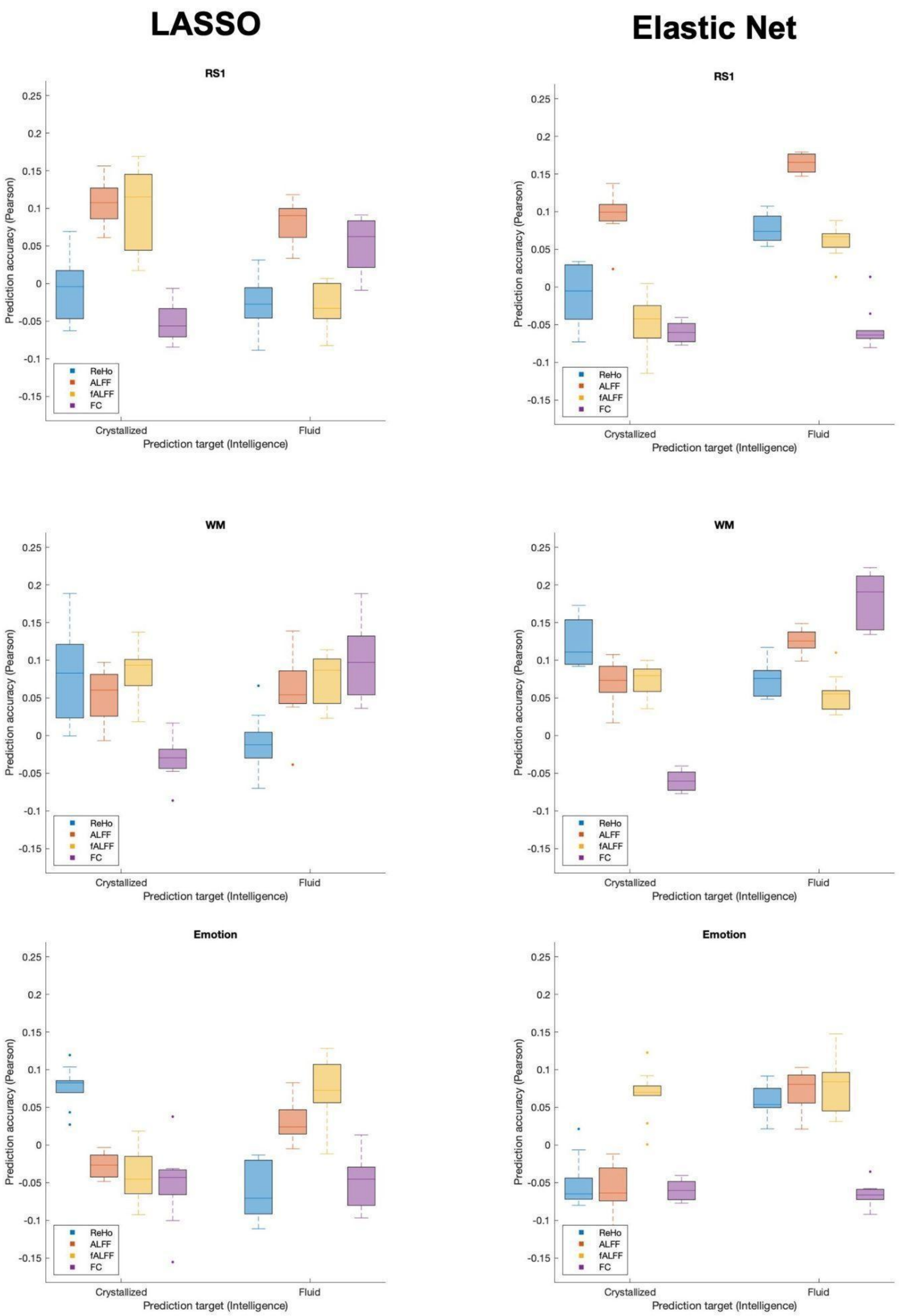
LASSO
Elastic Net
RS1
WM
Emotion
Prediction accuracy (Pearson)
Prediction target (Intelligence)
Crystallized
Fluid
ReHo
ALFF
fALFF
FC

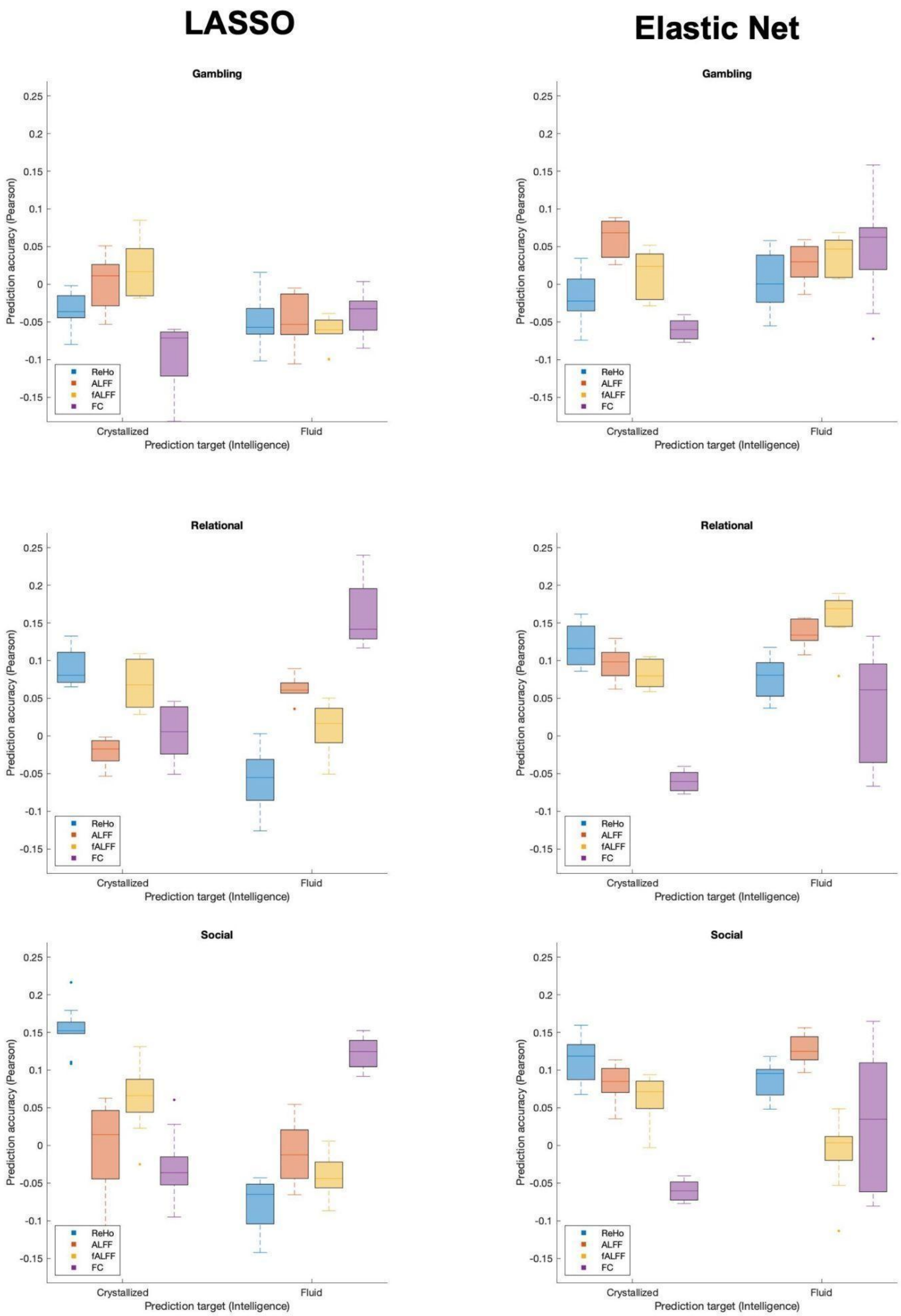
LASSO
Elastic Net
Gambling
Relational
Social
Prediction accuracy (Pearson)
Prediction target (Intelligence)
Crystallized
Fluid
ReHo
ALFF
fALFF
FC

Fig. S12: Prediction of crystallized and fluid intelligence using the fingerprints of the different sessions of the Human Connectome Project sample, corrected for age and brain volume. Each boxplot shows the prediction performance across five cross-validation runs. On the left, the LASSO algorithm was used; on the right, Elastic Net. *Asterisks indicate median cross-validation prediction accuracy (Pearson's r) > 0.15 across folds for both LASSO and Elastic Net models.

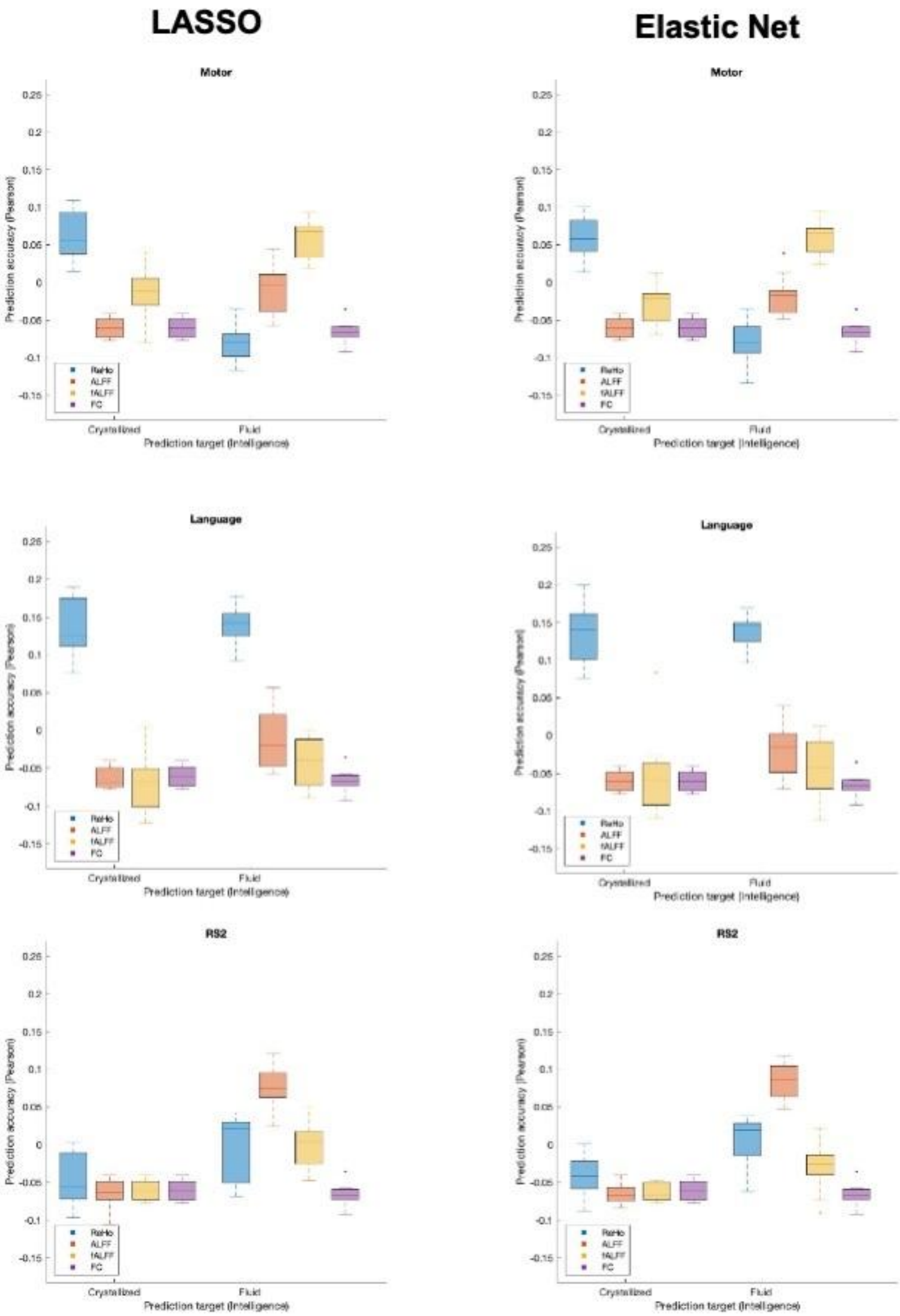
LASSO
Elastic Net
Motor
Language
RS2
Prediction accuracy (Pearson)
Prediction target (Intelligence)
Crystallized
Fluid
ReHo
ALFF
fALFF
FC

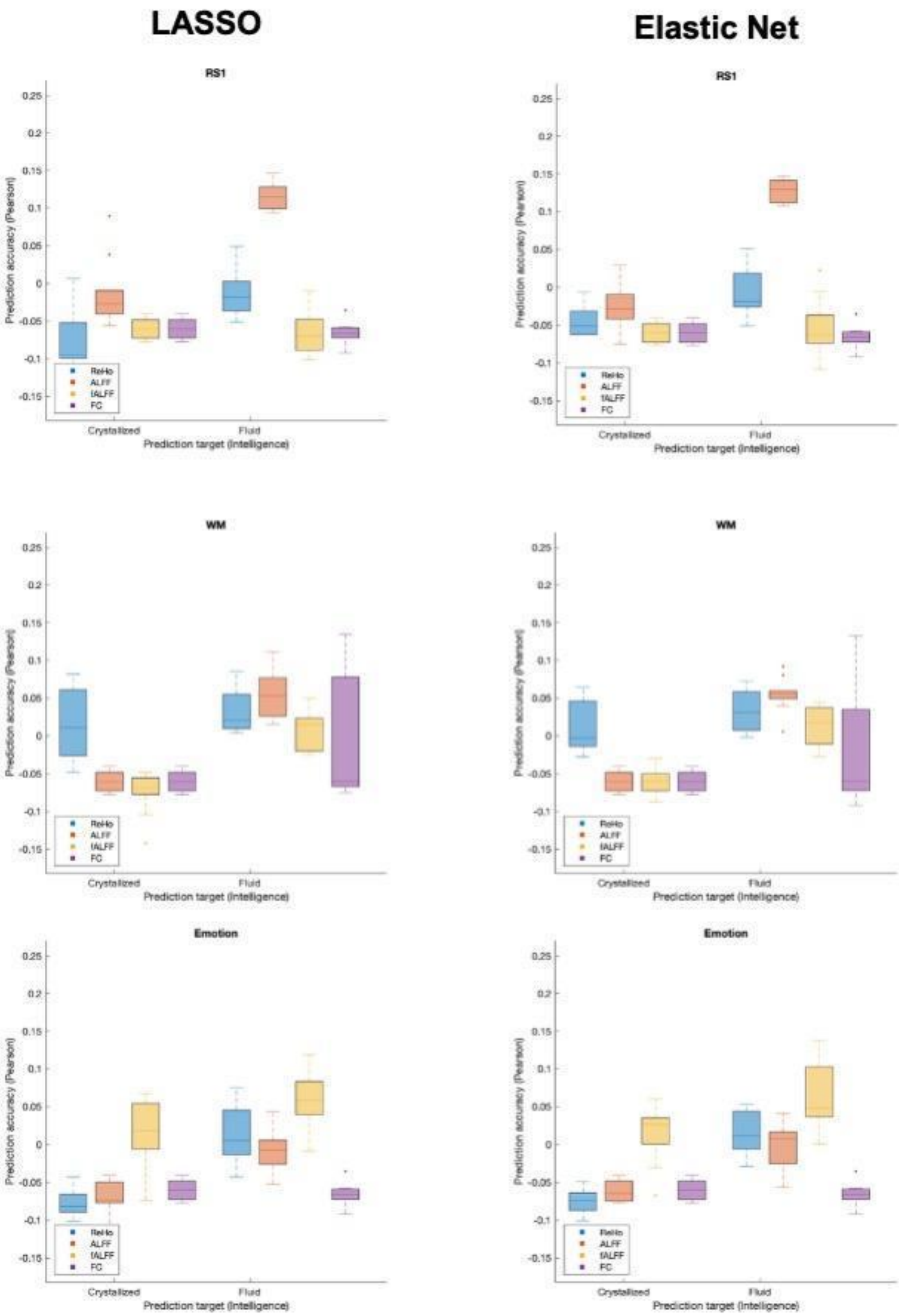
LASSO
Elastic Net
RS1
WM
Emotion
Prediction accuracy (Pearson)
Prediction target (Intelligence)
Crystallized
Fluid
ReHo
ALFF
fALFF
FC

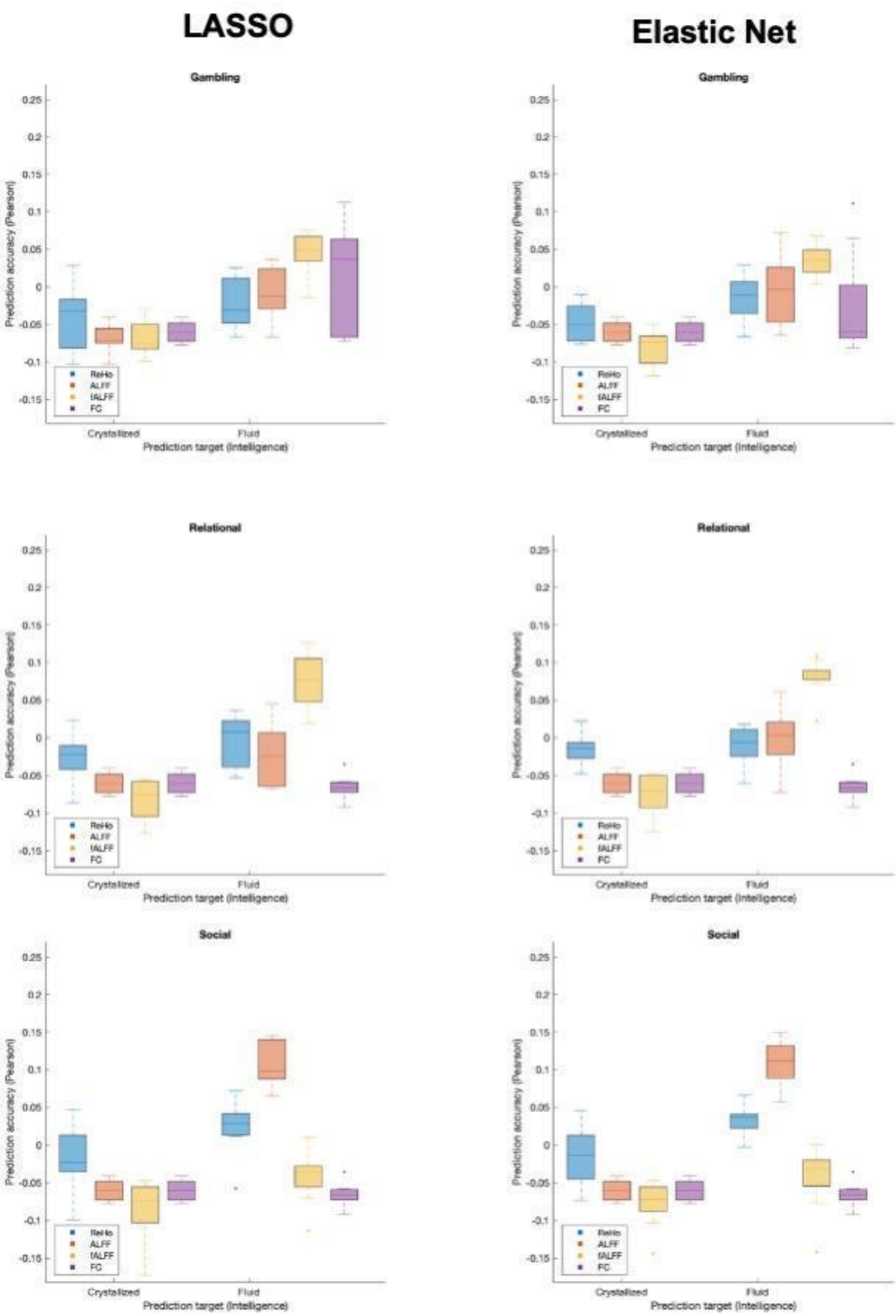

Fig. S13: Prediction of crystallized and fluid intelligence using the fingerprints of the different sessions of the Human Connectome Project sample, corrected for age, brain volume and average framewise displacement (FD). Each boxplot shows the prediction performance across five cross-validation runs. On the left, the LASSO

algorithm was used; on the right, Elastic Net. *Asterisks indicate median cross-validation prediction accuracy (Pearson's r) > 0.15 across folds for 1) both LASSO and Elastic Net models, 2) with (Fig. S13) and without correction for average FD (Fig. S12).

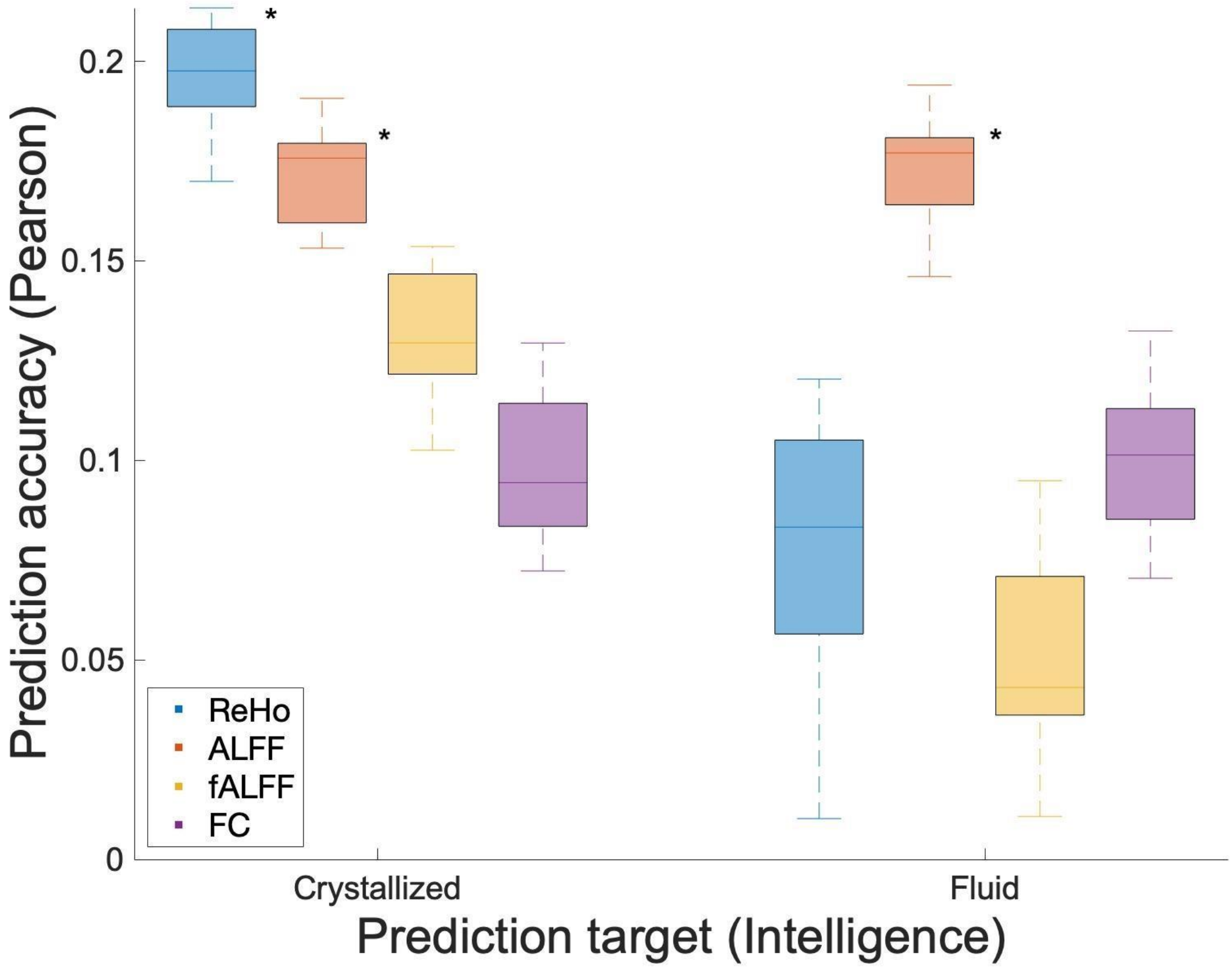

Fig. S14: Prediction of crystallized and fluid intelligence using the stability of fingerprints of the nine sessions of the Human Connectome Project sample, corrected for age and brain volume. Each boxplot shows the prediction performance across five cross-validation runs using Elastic Net. Abbreviations: ReHo = regional homogeneity; ALFF = amplitude of low-frequency fluctuations; fALFF = fractional ALFF; FC = functional connectome. *Asterisks indicate median cross-validation prediction accuracy (Pearson's r) > 0.15 across folds.

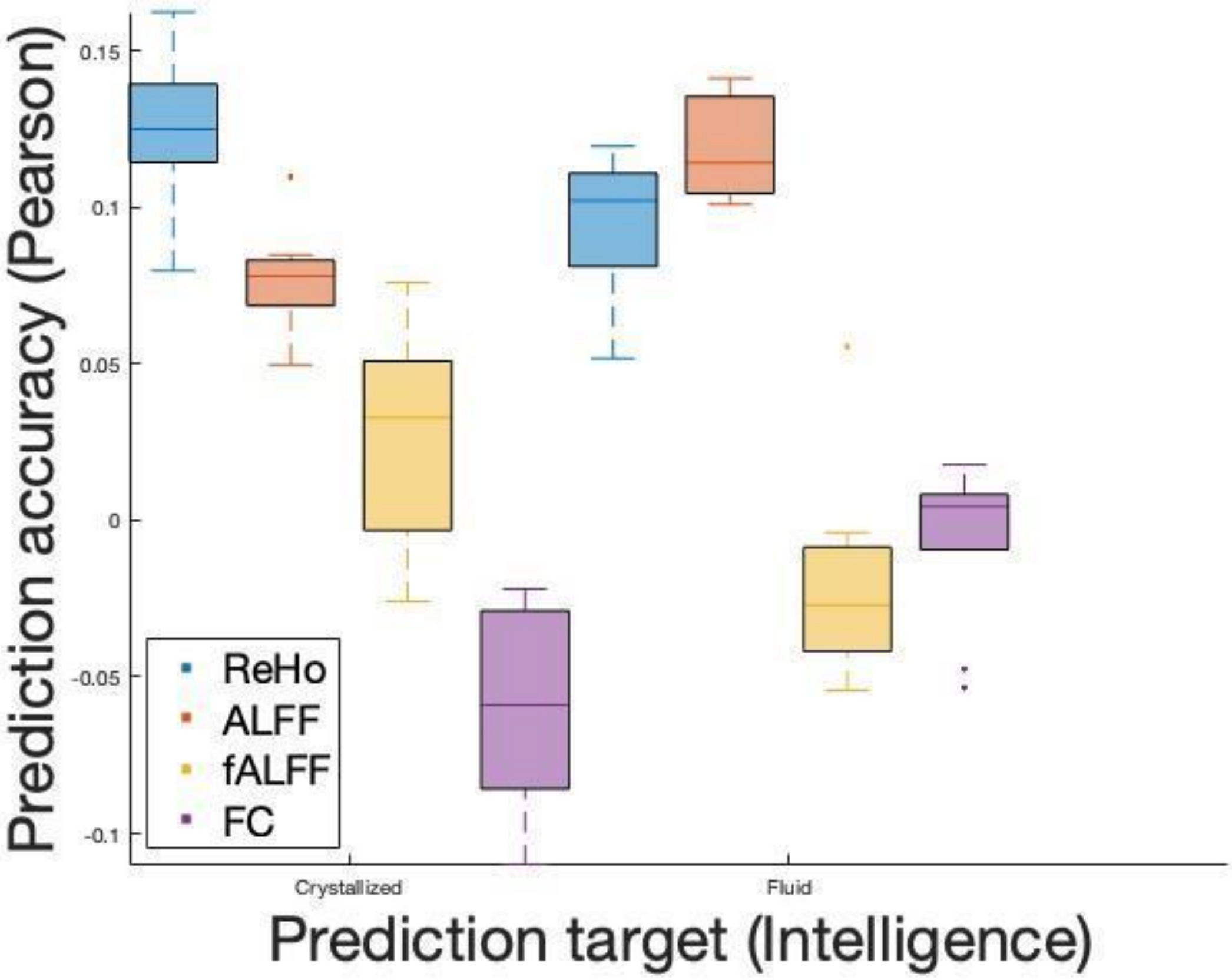

Fig. S15: Prediction of crystallized and fluid intelligence using the stability of fingerprints of the nine sessions of the Human Connectome Project sample, corrected for age, brain volume, and framewise-displacement. Each boxplot shows the prediction performance across five cross-validation runs using Elastic Net. Abbreviations: ReHo = regional homogeneity; ALFF = amplitude of low-frequency fluctuations; fALFF = fractional ALFF; FC = functional connectome.

Correlations between intelligence and fingerprint stability in two clinical samples

Table S2: Correlations between subtests of fluid intelligence (Matrix Reasoning; 'WAIS_MATRICS_SCALE') or crystallized intelligence (Vocabulary; 'WAIS_VOCAB_SCALED') and fingerprint stability in the OpenNeuro.ds000115 sample.

	Healthy controls	Schizophrenia

	ReHo	ALFF	fALFF	FC	ReHo	ALFF	fALFF	FC
**Matrix Reasoning**	r=0.10, p=0.68	r=0.08, p=0.76	r=0.30, p=0.22	r=0.02, p=0.94	r=-0.50, p=0.03*	r=-0.65, p=0.002*	r=-0.16, p=0.49	r=-0.60, p=0.005*
**Vocabulary**	r=0.19, p=0.45	r=0.48, p=0.04*	r=-0.02, p=0.94	r=0.16, p=0.51	r=0.06, p=0.80	r=-0.22, p=0.35	r=0.04, p=0.86	r=-0.03, p=0.90

Table S3: Correlations between fluid cognition (composite score; 'nih_fluidcogcomp_unadjusted') or crystallized cognition (composite score; 'nih_crycogcomp_unadjusted'), and fingerprint stability in HCP Early Psychosis sample.

	Healthy controls				Non-affective psychosis			
	**ReHo**	**ALFF**	**fALFF**	**FC**	**ReHo**	**ALFF**	**fALFF**	**FC**
**Fluid cognition**	r=0.02, p=0.88	r=-0.02, p=0.87	r=0.01, p=0.93	r=-0.01, p=0.95	r=-0.03, p=0.79	r=-0.04, p=0.77	r=0.002, p=0.99	r=0.09, p=0.46
**Crystallized cognition**	r=-0.06, p=0.68	r=-0.01, p=0.96	r=0.18, p=0.22	r=0.11, p=0.44	r=-0.02, p=0.88	r=0.01, p=0.91	r=0.06, p=0.65	r=0.16, p=0.18

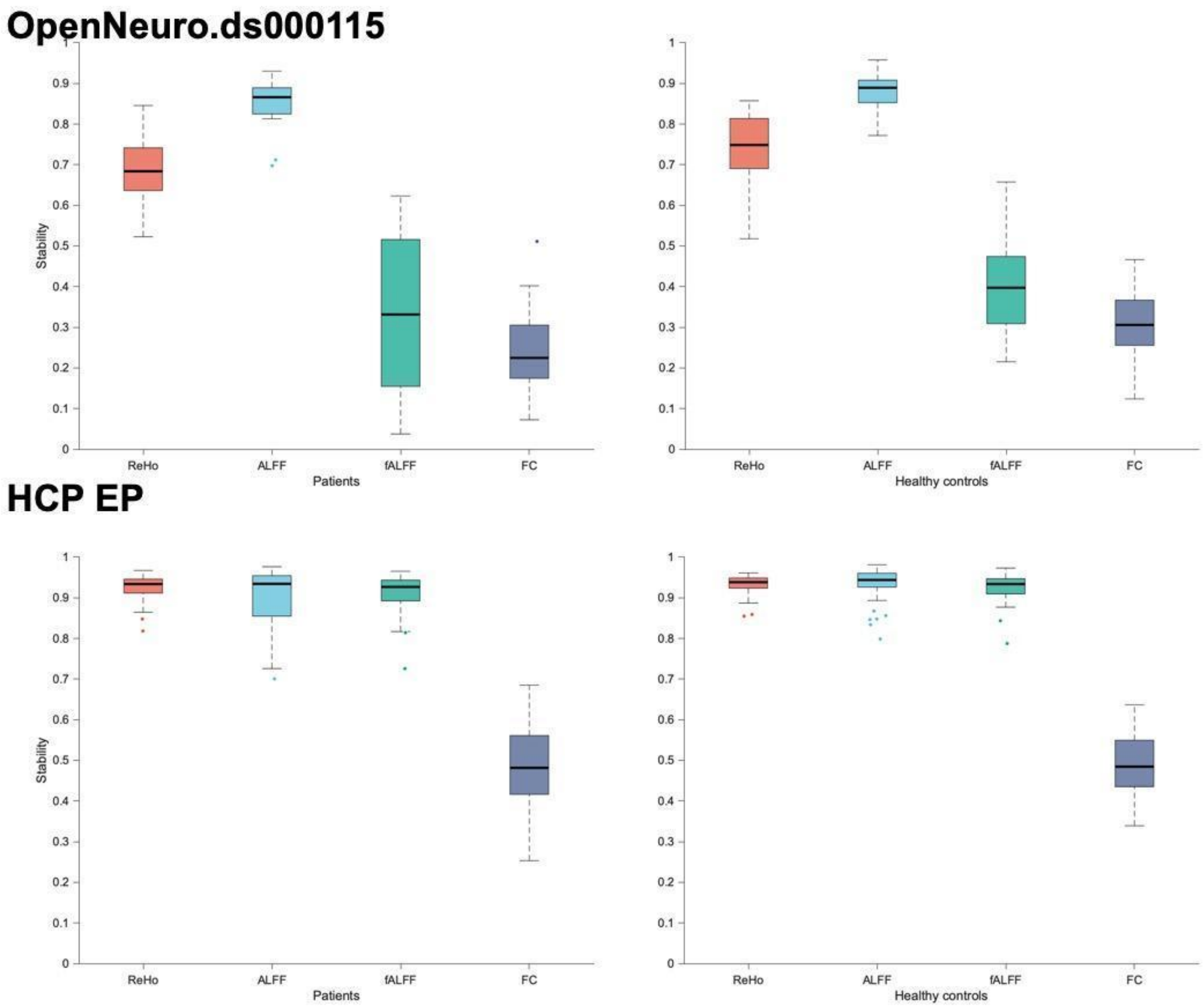

Fig. S16: stability of fingerprints. Abbreviations: HCP EP = Human Connectome Project Early Psychosis sample.

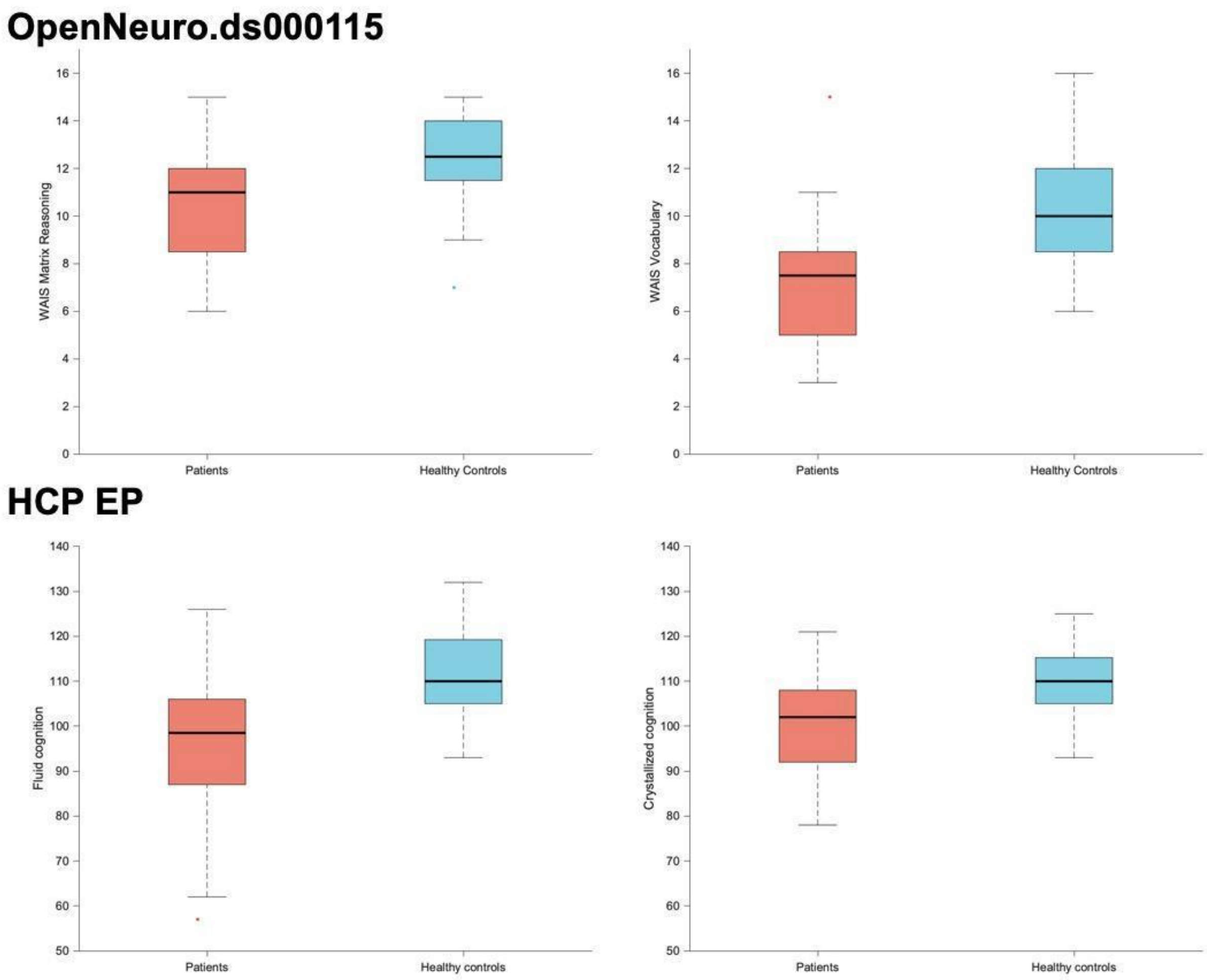

Fig. S17: variation in intelligence scores. Abbreviations: HCP EP = Human Connectome Project Early Psychosis sample.

References

Barch, D. M., Burgess, G. C., Harms, M. P., Petersen, S. E., Schlaggar, B. L., Corbetta, M., Glasser, M. F., Curtiss, S., Dixit, S., Feldt, C., Nolan, D., Bryant, E., Hartley, T., Footer, O., Bjork, J. M., Poldrack, R., Smith, S., Johansen-Berg, H., Snyder, A. Z., … WU-Minn HCP Consortium. (2013). Function in the human connectome: task-fMRI and individual differences in behavior. *Neuroimage*, *80*, 169–189. https://doi.org/10.1016/j.neuroimage.2013.05.033

Chang, W. (2014). *extrafont: Tools for using fonts* (0.17) [Computer manual].

Esteban, O., Markiewicz, C. J., Blair, R. W., Moodie, C. A., Isik, A. I., Erramuzpe, A., Kent, J. D., Goncalves, M., DuPre, E., Snyder, M., Oya, H., Ghosh, S. S., Wright, J., Durnez, J., Poldrack, R. A., & Gorgolewski, K. J. (2019). fMRIPrep: a robust preprocessing pipeline for functional MRI. *Nature Methods*, *16*(1), 111–116. https://doi.org/10.1038/s41592-018-0235-4

Finn, E. S., Shen, X., Scheinost, D., Rosenberg, M. D., Huang, J., Chun, M. M., Papademetris, X., & Constable, R. T. (2015). Functional connectome fingerprinting: identifying individuals using patterns of brain connectivity. *Nature Neuroscience*, *18*(11), 1664–1671. https://doi.org/10.1038/nn.4135

Fischl, B. (2012). FreeSurfer. *Neuroimage*, *62*(2), 774–781. https://doi.org/10.1016/j.neuroimage.2012.01.021

Glasser, M. F., Sotiropoulos, S. N., Wilson, J. A., Coalson, T. S., Fischl, B., Andersson, J. L., Xu, J., Jbabdi, S., Webster, M., Polimeni, J. R., Van Essen, D. C., Jenkinson, M., & WU-Minn HCP Consortium. (2013). The minimal

preprocessing pipelines for the Human Connectome Project. *Neuroimage*, *80*, 105–124. https://doi.org/10.1016/j.neuroimage.2013.04.127

Jenkinson, M., Bannister, P., Brady, M., & Smith, S. (2002). Improved optimization for the robust and accurate linear registration and motion correction of brain images. *Neuroimage*, *17*(2), 825–841. https://doi.org/10.1006/nimg.2002.1132

Jenkinson, M., Beckmann, C. F., Behrens, T. E., Woolrich, M. W., & Smith, S. M. (2012). FSL. *Neuroimage*, *62*(2), 782–790. https://doi.org/10.1016/j.neuroimage.2011.09.015

Kong, R., Li, J., Orban, C., Sabuncu, M. R., Liu, H., Schaefer, A., Sun, N., Zuo, X.-N., Holmes, A. J., Eickhoff, S. B., & Yeo, B. T. T. (2019). Spatial Topography of Individual-Specific Cortical Networks Predicts Human Cognition, Personality, and Emotion. *Cerebral Cortex*, *29*(6), 2533–2551. https://doi.org/10.1093/cercor/bhy123

Nooner, K. B., Colcombe, S. J., Tobe, R. H., Mennes, M., Benedict, M. M., Moreno, A. L., Panek, L. J., Brown, S., Zavitz, S. T., Li, Q., Sikka, S., Gutman, D., Bangaru, S., Schlachter, R. T., Kamiel, S. M., Anwar, A. R., Hinz, C. M., Kaplan, M. S., Rachlin, A. B., … Milham, M. P. (2012). The NKI-Rockland Sample: A Model for Accelerating the Pace of Discovery Science in Psychiatry. *Frontiers in Neuroscience*, *6*, 152. https://doi.org/10.3389/fnins.2012.00152

Repovš, G., & Barch, D. M. (2012). Working memory related brain network connectivity in individuals with schizophrenia and their siblings. *Frontiers in Human Neuroscience*, *6*, 137. https://doi.org/10.3389/fnhum.2012.00137

Shen, X., Tokoglu, F., Papademetris, X., & Constable, R. T. (2013). Groupwise whole-brain parcellation from resting-state fMRI data for network node identification. *Neuroimage*, *82*, 403–415. https://doi.org/10.1016/j.neuroimage.2013.05.081

Venkatesh, M., Jaja, J., & Pessoa, L. (2020). Comparing functional connectivity matrices: A geometry-aware approach applied to participant identification. *Neuroimage*, *207*, 116398. https://doi.org/10.1016/j.neuroimage.2019.116398

Wickham, H. (2007). Reshaping Data with the reshape Package. *Journal of Statistical Software*, *21*(12). https://doi.org/10.18637/jss.v021.i12

Wickham, H. (2016). *ggplot2: Elegant Graphics for Data Analysis*. Springer-Verlag New York.

Woolrich, M. W., Ripley, B. D., Brady, M., & Smith, S. M. (2001). Temporal autocorrelation in univariate linear modeling of FMRI data. *Neuroimage*, *14*(6), 1370–1386. https://doi.org/10.1006/nimg.2001.0931

Zang, Y., Jiang, T., Lu, Y., He, Y., & Tian, L. (2004). Regional homogeneity approach to fMRI data analysis. *Neuroimage*, *22*(1), 394–400. https://doi.org/10.1016/j.neuroimage.2003.12.030
